# Integrative analyses of transcriptome sequencing identify novel functional lncRNAs in esophageal squamous cell carcinoma

**DOI:** 10.1038/oncsis.2017.1

**Published:** 2017-02-13

**Authors:** C-Q Li, G-W Huang, Z-Y Wu, Y-J Xu, X-C Li, Y-J Xue, Y Zhu, J-M Zhao, M Li, J Zhang, J-Y Wu, F Lei, Q-Y Wang, S Li, C-P Zheng, B Ai, Z-D Tang, C-C Feng, L-D Liao, S-H Wang, J-H Shen, Y-J Liu, X-F Bai, J-Z He, H-H Cao, B-L Wu, M-R Wang, D-C Lin, H P Koeffler, L-D Wang, X Li, E-M Li, L-Y Xu

**Affiliations:** 1 The Key Laboratory of Molecular Biology for High Cancer Incidence Coastal Chaoshan Area, Shantou University Medical College, Shantou, China; 2 School of Medical Informatics, Daqing Campus, Harbin Medical University, Daqing, China; 3 Shantou Central Hospital, Affiliated Shantou Hospital of Sun Yat-sen University, Shantou, China; 4 College of Bioinformatics Science and Technology, Harbin Medical University, Harbin, China; 5 Cancer Institute/Hospital, Peking Union Medical College and Chinese Academy of Medical Sciences, Beijing, China; 6 Division of Hematology/Oncology, Cedars-Sinai Medical Center, University of California, Los Angeles School of Medicine, Los Angeles, CA, USA; 7 Cancer Science Institute of Singapore, National University of Singapore, Singapore, Singapore; 8 National University Cancer Institute of Singapore, National University Health System and National University Hospital, Singapore, Singapore; 9 Henan Key Laboratory for Esophageal Cancer Research of The First Affiliated Hospital, Zhengzhou University, Zhengzhou, China

## Abstract

Long non-coding RNAs (lncRNAs) have a critical role in cancer initiation and progression, and thus may mediate oncogenic or tumor suppressing effects, as well as be a new class of cancer therapeutic targets. We performed high-throughput sequencing of RNA (RNA-seq) to investigate the expression level of lncRNAs and protein-coding genes in 30 esophageal samples, comprised of 15 esophageal squamous cell carcinoma (ESCC) samples and their 15 paired non-tumor tissues. We further developed an integrative bioinformatics method, denoted URW-LPE, to identify key functional lncRNAs that regulate expression of downstream protein-coding genes in ESCC. A number of known onco-lncRNA and many putative novel ones were effectively identified by URW-LPE. Importantly, we identified lncRNA625 as a novel regulator of ESCC cell proliferation, invasion and migration. ESCC patients with high lncRNA625 expression had significantly shorter survival time than those with low expression. LncRNA625 also showed specific prognostic value for patients with metastatic ESCC. Finally, we identified E1A-binding protein p300 (EP300) as a downstream executor of lncRNA625-induced transcriptional responses. These findings establish a catalog of novel cancer-associated functional lncRNAs, which will promote our understanding of lncRNA-mediated regulation in this malignancy.

## Introduction

Esophageal squamous cell carcinoma (ESCC) is one of the more prevalent and lethal cancers worldwide.^1, 2^ In eastern Asia, ESCC is associated with high morbidity and mortality compared with Western countries.^1, 2^ To date, ESCC-related research has primarily focused on the deregulation of protein-coding genes (PCGs) and microRNAs to identify oncogenes and tumor suppressors, thereby missing long non-coding RNAs (lncRNAs).^3, 4^ LncRNAs are an RNA species >200 bp in length and expressed in a tissue-specific manner. Several well-described examples have shown that lncRNAs have critical roles in cancer initiation and progression, and thus may mediate oncogenic or tumor suppressing effects, as well as comprise a new class of cancer therapeutic targets.^5, 6, 7^ Examples include the increased expression of HOTAIR in metastatic breast cancer,^5^ oncogenicity and tumor-suppressive properties of H19 in different cancers,^6^ ANRIL-induced epigenetic silencing of p15 in leukemia,^7^ and the ability of MALAT1 to confer high metastatic potential in non-small cell lung cancer.^8^ In contrast to these well-described examples, little is known about the functions of most lncRNAs in caner initiation and progression. For example, ESCCAL-1 was found to be an onco-lncRNA in esophageal cancer development, and high expression of BC200 or MALAT1 has been shown to be a novel predictive marker for ESCC patients who received radical resection.^9, 10, 11^ Overall, a handful of lncRNAs have documented roles in ESCC.^4, 9, 10, 11, 12, 13, 14, 15, 16, 17^

Next-generation transcriptome sequencing (RNA-seq) has provided a method to delineate the entire set of transcriptional aberrations in a disease, including lncRNAs and PCGs. For example, using RNA-seq to analyze prostate cancer tissues, the landscape of lncRNAs in prostate cancer has been recently defined and notably includes prostate cancer functional lncRNAs, such as PCA1^18^ and SChLAP1.^19^ In the case of ESCC, Ma *et al.*^20^ has applied transcriptome sequencing to ESCC tissues from three patients and adjacent non-tumor tissues. However, because the existing transcriptome sequencing for ESCC focuses on research of PCGs, most of the functional lncRNAs in ESCC have yet to be identified. The functions of lncRNAs are closely associated with their abundance of transcripts and downstream target PCGs directly or indirectly regulated by them.^21^ LncRNA-induced transcriptional dysregulation of target PCGs has become an effective strategy to identify key functional lncRNAs and several successful methods have been developed.^11, 22, 23^ For example, a random walk strategy has been used to search for candidate prostate cancer-related lncRNAs in the lncRNA-PCG bipartite network based on sample correlation, and a lncRNA-PCG co-expression network has been constructed to predict the functions of lncRNAs.^22^ A limitation of these methods is the requirement for known cancer lncRNAs to serve as seeds. However, for many diseases such as ESCC, only a few known lncRNAs are available to be used as seeds, which decreases the predictive power for a disease. In addition, methods based on known lncRNAs as seeds also tend to identify neighborhood lncRNAs of well-studied cancer lncRNAs, and thus lack ability to identify novel functional lncRNAs.

Here, we perform RNA-seq to investigate expression levels of lncRNAs and PCGs in 30 esophageal samples (15 paired ESCC and non-tumor esophageal tissues). We further developed a method, denoted URW-LPE (for Unsupervised Random Walk method with each dysregulated LncRNA/PCG as a seed and extended co-Expression relation as an edge), to identify novel potential functional lncRNAs based on global lncRNA-PCG network information.

## Results

### Expression analysis of ESCC-related lncRNAs and coding genes

**Figure 1:**
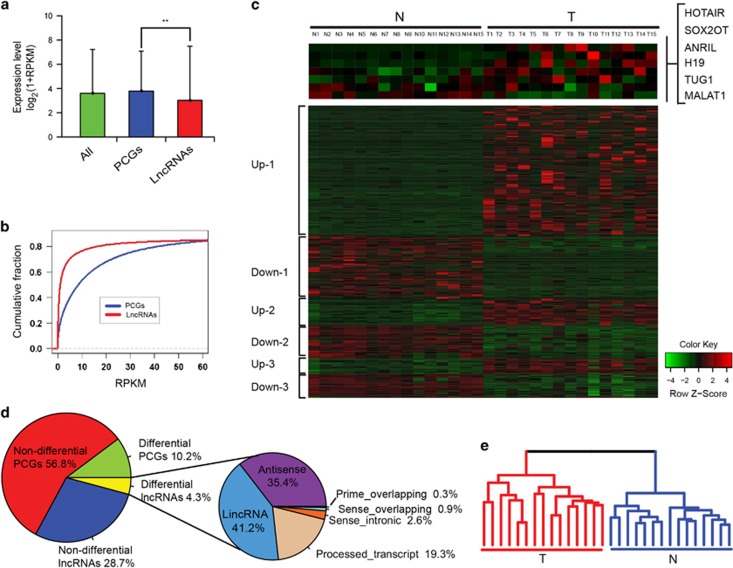
Transcriptome sequencing of ESCC. (**a**) Average expression level of lncRNAs and PCGs. (**b**) Line graph showing that lncRNAs are expressed less than PCGs. (**c**) Heat maps of the expression levels of lncRNAs that showed significant differential expression between cancer and normal ESCC tissues. (**d**) Global overview of lncRNAs and PCGs in ESCC. Left pie chart displays differential and non-differential lncRNA/PCG distribution in ESCC. Pie charts on the right display differentially expressed lncRNAs, respectively categorized as sense-intronic, lincRNA, antisense, sense-overlapping, prime overlapping and processed transcripts. (**e**) Hierarchical clustering analysis on the expression profile of 1226 differentially expressed lncRNAs. These lncRNAs were identified as differentially expressed when fold change >2 or <½, and a DESeq FDR value <0.25. The tree diagram exhibits a 2-branch partition with the 15 ESCC tumor samples clustered together and well separated from their matched non-tumor controls.

RNA-seq was performed on 30 esophageal samples (15 paired ESCC and non-tumor esophageal tissues, Supplementary Table 1) from Chinese patients. Sequencing reads were mapped against the human genome assembly (NCBI Build 37) using Tophat (v2.0.6). For each sample, on average, 62.30 million reads were mapped to known human genes (Supplementary Table 2). Congruent with previous reports, lncRNAs were expressed at levels lower than PCGs (Figure 1a), with expression levels of 75% lncRNAs being less than 10 RPKM (Figure 1b). We identified 1226 differentially expressed lncRNAs between tumors and non-tumor matched samples (Figure 1c; fold change >2 or <½, and a DESeq FDR value <0.25). These differentially expressed lncRNAs made up 4.3% of all expressed lncRNAs and PCGs, of which lncRNA made up 41.2% of all differential lncRNAs (Figure 1d). With this same criterion, we identified 2996 differentially expressed PCGs, of which 1834 were upregulated and 1162 were downregulated.

We focused on these differentially expressed lncRNAs and PCGs (fold change >2 or fold change <½, and a DESeq FDR value <0.25). Hierarchical clustering analysis on the expression profile of 1,226 differentially expressed lncRNAs exhibited a 2-branch partition with the 15 ESCC tumor samples clustered together and well separated from their matched non-tumor controls (Figure 1e). Similar results were obtained when using the 2,996 differentially expressed PCGs (Supplementary Figure 1). To validate the utility of the RNA-seq data to identify cancer-related lncRNAs, we examined the expression patterns of 8 literature-evidenced ESCC-related lncRNAs (top panel in Figure 1c). Notably, RNA-seq analysis revealed that six of 8 known ESCC-related lncRNAs showed a consistently upregulated expression pattern. Three known ESCC lncRNAs, comprising HOTAIR,^15^ ANRIL,^16^ and SOX2OT,^17^ showed statistically significantly differential expression between tumor and non-tumor samples (FDR values of DESeq were 5.51E-05, 0.0048 and 0.21, respectively). Results of expression at the exon level demonstrated that our RNA-seq was capable of correctly measuring expression of lncRNAs (Supplementary Figure 2). The upregulation of another three known ESCC lncRNAs, comprising PCA1,^24^ TUG1^25^ and H19,^26^ were also consistent with the upregulation reported by other groups.^24, 25, 26^ Interestingly, we found that two cancer-related lncRNAs PVT1^27^ and WT1-AS,^28^ which were not reported in ESCC, displayed differential expression based on our RNA-seq analysis. MIR31HG,^29^ a tumor-suppressive lncRNA known to be downregulated in glioblastoma, showed increased expression in ESCC, suggesting complex and context-dependent functions of lncRNAs in different cancer types. DLX6-AS1, LINC00162 and NPPA-AS1, which were reported to function in development, narcolepsy and modulation of blood pressure,^30, 31, 32^ showed differential expression between ESCC tissue samples and paired non-tumor tissues, suggesting their function in ESCC.

### Identification of functional lncRNAs in ESCC

**Figure 2:**
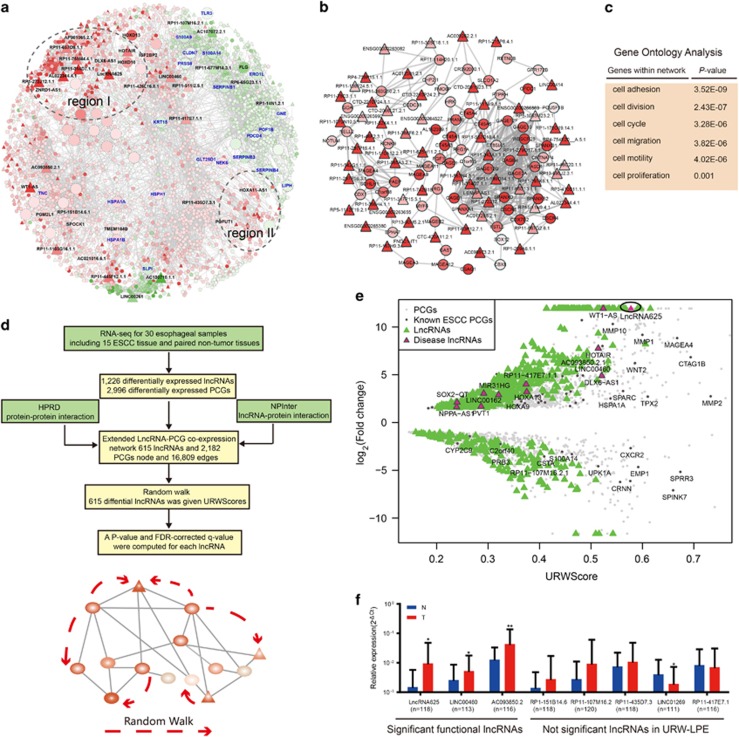
Identification of functional lncRNAs in ESCC via downstream target PCGs. (**a**) The extended lncRNA-PCG co-expression network. The network is displayed using Cytoscape software according to the ‘spring' layout. Node size is proportional to the degree of the node. Node color reflects differential expression level. Nodes with a zigzag border line are differential PCGs after lncRNA625 knockdown in the network. Interesting lncRNAs and PCGs are enlarged or labeled using their names. For example, nodes with a blue label are genes regulated by lncRNA625. Circles with dashed lines in the network represent subnetwork modules with many highly upregulated lncRNAs and PCGs. (**b**) An active subnetwork module in the lncRNA-PCG co-expression network. (**c**) PCGs in the lncRNA-PCG co-expression network are annotated to Gene Ontology to identify their functions. (**d**) Schematic overview of URW-LPE. The top figure represents the data stream of URW-LPE, and the bottom figure represents the running process of the random walk that is the core step of URW-LPE. (**e**) Fold change and URWScore value of each lncRNA in the lncRNA-PCG co-expression network. Known disease lncRNAs and ESCC PCGs are labeled. (**f**) Scatter plots of the relative expression levels of qRT–PCR of lncRNAs in an additional 120 paired ESCC patient samples. Note that because a lncRNA may not be identified by qRT–PCR in the corresponding sample, the number of actual samples with expression level for each lncRNA was slightly less than the total number of samples. Comparisons of the relative expression between tumor (T) and non-tumor (N) were performed using a paired *t*-test. A *P*-value <0.05 was considered statistically significant. Black horizontal lines are means with s.e.m.

LncRNAs do not encode protein, but often either directly or indirectly regulate transcription, splicing and translation of downstream target PCGs.^3^ These reports prompted us to test the correlation between the expression of lncRNA and PCG to pinpoint key lncRNAs. We identified 4,554 pair significant co-expression relationships between 615 differential lncRNAs and 2182 differential PCGs. We further constructed an extended lncRNA-PCG co-expression network based on differentially expressed lncRNAs/PCGs (Figure 2a). Edges in the network were constructed if two molecules were significantly co-expressed (FDR <1.0e-7) or they had direct protein-protein or lncRNA-protein interaction relationship in the HPRD or NPInter databases (Materials and methods section). In the final network, 615 lncRNAs and 2182 PCGs with 16 809 edges remained. As shown in Figure 2a, the upregulated nodes exceeded the downregulated nodes. Four hundred fifty (73.17%) lncRNAs and 1456 (66.72%) PCGs were upregulated in the network, suggesting that lncRNAs might stimulate downstream targets mainly through inducing mechanisms. Topological analysis showed that the network displayed a power-law distribution and module tendency (Supplementary Figure 3). Indeed, many lncRNAs and PCGs closely gathered together to form active modules (for example, regions I and II in Figure 2a). Network active analysis using the jActive method, a widely used active module mining method (http://www.cytoscape.org/), identified a highly active subnetwork module (Figure 2b) and the lncRNAs and PCGs in the module were mostly located in region I of the network. PCGs in the network were significantly associated with cancer-related functions (Figure 2c).

To identify more effectively the lncRNAs that are able to regulate the expression of downstream PCGs from our extended lncRNA-PCG co-expression network, we developed an unsupervised random walk, denoted URW-LPE, with each dysregulated lncRNA/PCG as a seed (Figure 2d; Materials and methods section). A lncRNA will obtain a high score when it is highly differential and closely associated with highly differential PCGs from the global network view. A total of 615 differential lncRNAs in the network received the URWScore. Multiple known oncogenic lncRNAs were highly ranked according to URWScore, compared with their FC value, validating the URW-LPE procedure (Table 1 and Figure 2e). A good example is HOTAIR, a well-characterized ESCC lncRNA. HOTAIR was ranked 35th, according to the URWScore, but was only ranked 93rd according to the FC value (Table 1). The lncRNA DLX6-AS1 was ranked 31st by URW-LPE, but only ranked 199th according to its FC value (Table 1). The lncRNA in lung cancer tissues is significantly higher compared with paired adjacent normal lung tissues.^31^ Moreover, DLX6-AS1 might be a novel therapeutic target for lung cancer patients because it appears to enhance cancer invasion and metastasis.^31^ Taken together, the top-ranked lncRNAs, identified by URW-LPE, have potential to have novel functional roles in ESCC. Although the oncogenic lncRNA ANRIL was ranked 241st by URW-LPE, its rank in URW-LPE increased more than 100 compared with the rank according to FC value (Table 1). The higher rank of this lncRNA, given by URW-LPE, shows that our method has the ability for recalling functional lncRNAs in ESCC.

To comprehensively identify statistically significant lncRNAs, FDR-corrected *P*-values for each lncRNA in the network were calculated by URW-LPE through comparison of the URWScore, of each lncRNA, with that of background distribution. With a strict cutoff of FDR-corrected *P*-values <0.05 (corresponding to original *P*-values <0.005), URW-LPE identified 63 statistically significant lncRNAs (Supplementary Table 3). Of these lncRNAs, several including HOTAIR,^12, 13, 14, 15^ DLX6-AS1,^31^ AC130710.1.1^32^ and WT1-AS,^28^ are known functional lncRNAs in cancer. For the most well-characterized lncRNA HOTAIR, the URW-LPE analysis yielded a *P*-value of 0.0015 (corrected to 0.025 by FDR) (Table 1). In addition, DLX6-AS1,^31, 33^ AC130710.1.1,^32^ and WT1-AS^28^ have established functions in development,^33^ lung cancer,^31^ gastric cancer^32^ and acute myeloid leukemia^28^ (Table 1). With a cutoff of FDR-corrected *P-*values <0.1 (corresponding to original *P*-values <0.023), 147 significant lncRNAs were identified (Supplementary Table 3). For a downregulated functional lncRNA LINC00261,^34^ the URW-LPE yielded a *P*-value of 0.00047 (corrected to 0.017 by FDR) (Table 1). Jiang et al. demonstrated that LINC00261 enhances FOXA2 activation by facilitating SMAD2/3 recruitment to the FOXA2 promoter, and overexpression of FOXA2 is able to rescue endoderm differentiation defects.^34^

Finally, we focused on novel lncRNAs not reported by the existing studies to be associated with disease. From the statistically significant lncRNAs in URW-LPE (FDR-corrected *P-*values <0.05), three novel candidate upregulated lncRNAs (lncRNA625, LINC00460 and AC093850.2) with high URWScores and differential expression levels were selected for testing. We investigated the expression level of candidate lncRNAs in 120 paired ESCC tissues by PCR, quantitative reverse transcriptase–PCR (qRT–PCR) analysis (Supplementary Tables 4 and 5). According to scatter plots, the expression trends of all three lncRNAs were consistent with results of RNA-seq and were significantly upregulated in ESCC samples (paired*t*-test; Figure 2f). For most of lncRNAs not identified as significant by URW-LPE (FDR-corrected *P-*values >0.1), statistically significant differences were not obtained (paired *t*-test; Figure 2f), although the expression trends of lncRNAs were consistent with results of RNA-seq. These results demonstrate that novel functional lncRNAs in ESCC identified as statistically significant by URW-LPE can be confirmed by low-throughput experiment such as qRT–PCR.

### LncRNA625 modulates cancer cell proliferation, invasion and migration via affecting downstream target PCGs

**Figure 3:**
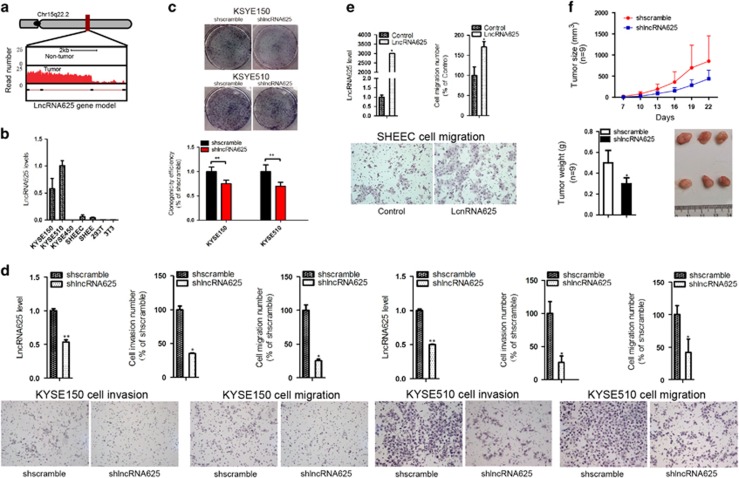
LncRNA625 modulates cancer cell proliferation, invasion and migration via affecting downstream target PCGs. (**a**) Read distributions of the RNA-seq gene model. (**b**) LncRNA625 expression in various human ESCC cells. (**c**) Colony formation of stably transfected KYSE150 and KYSE510 stained with haematoxylin solution after incubation for 15 days. (**d**) Invasion and migration of KYSE150 and KYSE510 cells stably transfected with shRNA against either lncRNA625 or with a scrambled RNA. (**e**) Migration of SHEEC cells detected at 48 h following transfection with either lncRNA625 expression vector or control vector, and detection of lncRNA625 levels by real-time RT-PCR. Values are mean±s.e.m. (**f**) LncRNA625 downregulation inhibited the proliferation of esophageal cancer cells. 1 × 10^6^ KYSE150-shlncRNA625 or shscramble cells were subcutaneously inoculated in the right flank of each BALB/c mouse (nu/nu) (*n*=9) and after one week, tumor volumes were measured every two days according to the formula: V=ab^2^/2 (‘a' represents the length of tumor tissue and ‘b' represents the width of tumor tissue). The average weight of tumors was determined after mice were euthanized by CO_2_ inhalation.

From these three lncRNAs, lncRNA625 displayed a high degree of differential expression and a high URWScore (Figures 2e and 3a), and it was selected for further functional characterization. LncRNA625 expression was measured in various human esophageal cancer cell lines and was found to be highly expressed in KYSE150 and KYSE510 ESCC cells (Figure 3b). To delineate its biological functions, endogenous lncRNA625 was downregulated in KYSE150 and KYSE510 cells, and colony formation assays were performed. Importantly, we observed that proliferation of KYSE150 and KYSE510 cells was reduced by expressing a short hairpin RNA against lncRNA625 (Figure 3c). Cell invasion and migration was also inhibited following silencing of lncRNA625 (Figure 3d). Similar results were obtained by small interfering RNAs (siRNA)-mediated knockdown approaches (Supplementary Figure 4). Conversely, ectopic expression of lncRNA625 in KYSE510 and SHEEC ESCC cells resulted in enhanced migration (Figure 3e). An *in vivo* tumorigenicity study in mice showed that the average tumor volumes of stably transfected KYSE150-shlncRNA625 cells was generally lower than control (Figure 3f, top panel). To further verify the results, we measured tumor weight and found that the average weight of tumors derived from stably transfected KYSE150-shlncRNA625 cells was less than control (Figure 3f, bottom panel). Taken together, these results indicate that lncRNA625 modulates cancer cell proliferation, invasion and migration.

**Figure 4:**
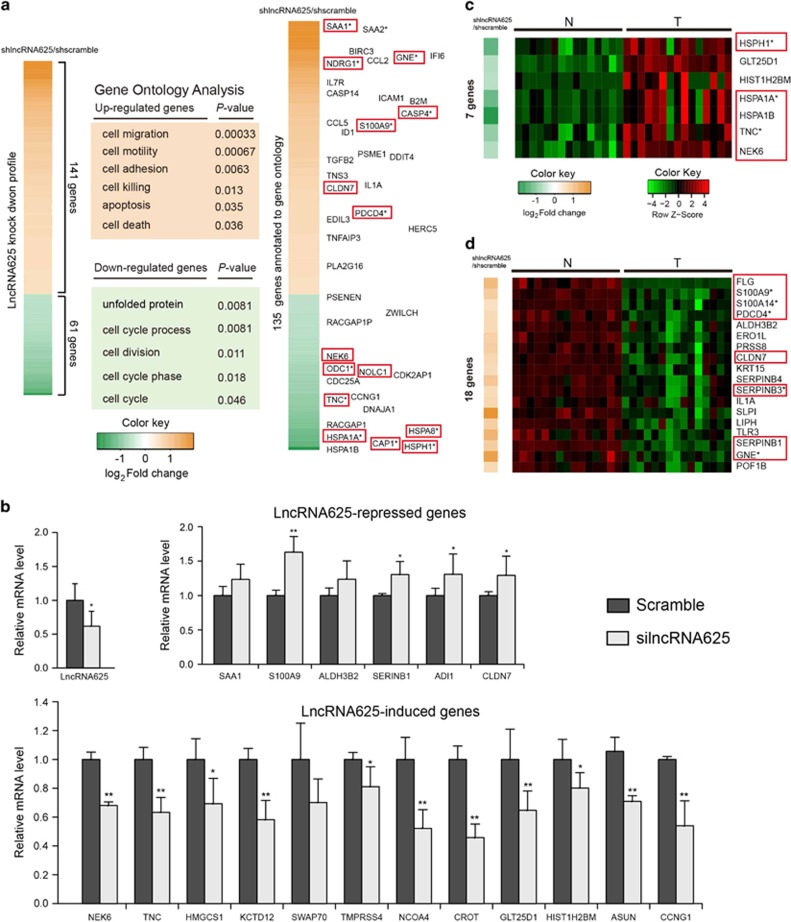
Gene expression profile analysis after lncRNA625 knockdown. (**a**) Gene expression profile analysis performed after lncRNA625 knockdown in cells stably transfected with either shlncRNA625 or scrambled shRNA (shscramble). (**b**) qRT–PCR of a representative panel of genes in scrambled and silncRNA625 (error bars are s.d., *n*=6). (**c**) PCGs downregulated by lncRNA625 knockdown and significantly upregulated in RNA-seq samples. (**d**) PCGs upregulated and downregulated, following lncRNA625 silencing, in RNA-seq samples. Genes boxed in red are literature-evidenced cancer-related genes. Genes with an asterisk are literature-evidenced ESCC-related genes.

Gene expression profiling by cDNA microarray analysis of the lncRNA625 knockdown KYSE150 cell line indicated that lncRNA625 knockdown affected the expression of 202 genes (141 up- and 61 downregulated; |log (fold change)|>log_2_1.5) (Figure 4a). In agreement with a potential role of lncRNA625 in regulating cell invasion and migration, gene ontology analysis of the differentially expressed genes showed preferential enrichment for cellular processes such as cell migration, cycle, motion and adhesion (Figure 4a).

We next focused on exploring cancer-related functional target genes regulated by lncRNA625, which were related to genes in GO terms, such as cell invasion and migration (Figure 4a, right). Most of the genes regulated by lncRNA625 were highly associated with cancer cell proliferation, invasion and migration (genes boxed in red in Figure 4a). Use of qRT–PCR for representative genes from the cDNA microarrays confirmed their dysregulation of expression (Figure 4b). Of note, many of the genes induced by lncRNA625, including NEK6,^35, 36, 37, 38^ TNC,^39, 40^ CCNG1,^41^ HIST1H2BM,^42^ NCOA4^43^ and KCTD12,^44^ have known oncogenic properties. Similarly, known tumor suppressors, such as SAA1,^45^ S100A9,^46, 47^ ADI1^48^ and CLDN7,^49^ were consistently upregulated after lncRNA625 knockdown (Figure 4b). Moreover, most of these genes displayed consistent expression pattern in the RNA-seq data (Figures 4c and d). That is, those upregulated (downregulated) PCGs, following lncRNA625 knockdown, were significantly downregulated (upregulated) in the RNA-seq sample (*P*-value=1.24e^−06^ and 0.041, respectively, hypergeometric test). Taken together, the above analyses, based on high-throughput gene expression profiling and low-throughput experiments, demonstrate that lncRNA-625 controls the up- and downregulation of multiple target PCGs to promote cancer cell proliferation, invasion and migration.

### LncRNA625 interacts with EP300 to regulate transcription of downstream target genes

**Figure 5:**
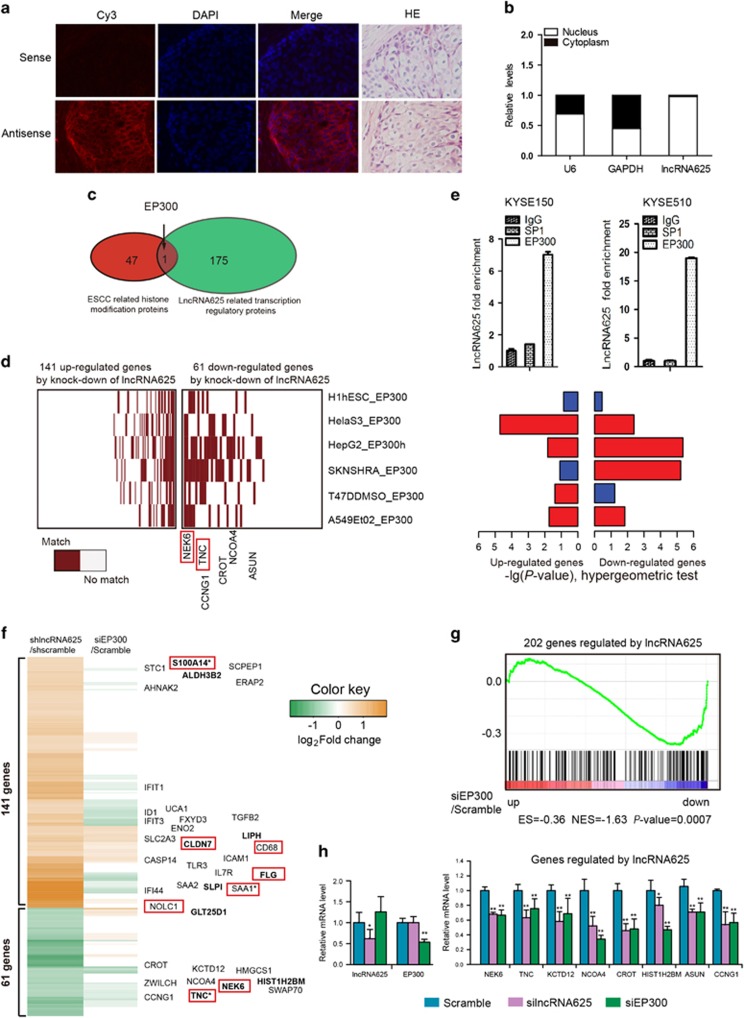
LncRNA625 interacts with EP300 to regulate downstream target genes. (**a**) LncRNA625 is located in the cytoplasm and nucleus of tumor tissue. Sense or antisense probe for lncRNA625 FISH were synthesized by *in vitro* transcription of T7 RNA polymerase, and 3 μm serial slides of ESCC tissues were hybridized with sense or antisense probes conjugated with biotin. Subsequently, the biotin signal was determined with Cy3-conjugated streptavidine. DAPI staining for was for nuclei, and haematoxylin and eosin staining was for tumor histomorphology. Scale bar: 40 ×. (**b**) Cytoplasmic and nuclear RNAs were isolated from KYSE510 cells, and lncRNA625 was detected by real-time RT–PCR. Levels of U6 snRNA (nuclear control transcript) and GAPDH (cytoplasmic control transcript) were detected by real-time RT–PCR. Values are mean±s.e. (**c**) Venn diagram showing the overlap between ESCC-related histone modification proteins and lncRNA625-related transcription regulatory proteins. (**d**) Comparison of 202 differentially expressed genes following silencing lncRNA625 in KYSE150 cells vs. a compendium of UCSC-published EP300 occupancy profiles in diverse cell types. (**e**) LncRNA625 interacts with EP300. RNA immunoprecipitation assays for EP300 were performed and RNA was extracted with 1 ml TRIzol, and lncRNA625 was detected by real-time RT-PCR in both KYSE150 and KYSE510 ESCC cells. IgG and SP1 were used as negative controls in the experiment. (**f**) Gene expression profile analysis was performed after either lncRNA625 or EP300 knockdown in KYSE 150 cells. Genes with |log_2_FC|>log_2_1.5, after lncRNA625 knockdown, are displayed in the heat map. (**g**) GSEA plot showing that genes regulated by lncRNA625 were enriched in the expression profile after knocking down EP300. In particular, those genes downregulated after knocking down EP300 received a high enrichment score. (**h**) qRT–PCR of a representative panel of genes in scrambled, silncRNA625 and siEP300 cells (error bars are s.d., *n*=6). Genes boxed in red are literature-evidenced cancer-related genes. Genes with an asterisk are literature-evidenced ESCC-related genes.

To determine the mechanism by which lncRNA625 regulates the transcription of downstream target PCGs, we initially localized lncRNA625 in ESCC tissue and showed that lncRNA625 was located in both nucleus and cytoplasm of tumor cells (Figure 5a). For KYSE510 cell lines, lncRNA625 was predominantly localized in the nucleus of cells (Figure 5b), suggesting that lncRNA625 could regulate transcription of downstream target genes through binding proteins in the nuclear chromatin. Thus, we focused on 48 histone modification related proteins reported to be highly associated with ESCC.^50^ LncRNA625-regulated PCGs were potentially regulated by 176 transcription-related regulatory proteins based on transcription protein analysis using the DAVID tool.^51^ Interestingly, of these 48 ESCC histone modification proteins, only the E1a-binding protein p300 (EP300) appeared in 176 lncRNA625-related transcription regulatory proteins (Figure 5b). Searching the UCSC genome browser, showed that EP300 occupancy frequently appeared in the promoter regions of multiple lncRNA625-regulated PCGs including NEK6, TNC, NCOA4 and CROT (Supplementary Figure 5). Furthermore, all 202 lncRNA625-regulated genes were compared with a compendium of published EP300 occupancy profiles in diverse cell types in the UCSC database (Supplementary Table 6). In several cancer cell lines, such as HeLa, HepG and SkNSHRA, genes downregulated by lncRNA625 knockdown were enriched for the endogenous EP300 occupancy pattern, but not enriched in hESC (Figure 5d). Compared with the upregulated genes following lncRNA625 knockdown, downregulated genes displayed higher statistical significance scores for EP300 occupancy in cancer cell lines (Figure 5d, hypergeometric test).

To explore EP300 binding to lncRNA625, catRAPID (http://big.crg.cat/gene_function_and_evolution/services/catrapid), a predictor of protein-RNA binding, was employed to assess the likelihood of protein-RNA interaction.^52^ LncRNA625 (nucleotide positions [nt] 40–140) and EP300 protein (amino acid residues 900–1600) were predicted to interact (Supplementary Figure 6). Next, we examined the binding of lncRNA625 and EP300 through RNA immunoprecipitation (RIP) in human KYSE150 and KYSE510 cells. LncRNA625 co-precipitated with EP300 in both cell lines (Figure 5e), suggesting that the interaction of lncRNA625 with EP300 mediates the transcription of target genes.

Secondly, EP300 was silenced and the expression of lncRNA625 target genes was examined. Gene expression profiling of knockdown samples on cDNA microarrays indicated that EP300 affected expression of several downstream lncRNA625 target genes (Figure 5f). Moreover, Gene Set Enrichment Analysis (GSEA)^53^ showed that genes regulated by lncRNA625 were enriched in the expression profile after knocking down EP300 (Figure 5g). Especially, those downregulated genes after knocking down EP300 received a high enrichment score (ES), suggesting that lncRNA625 might induce transcriptional responses of genes through interacting with EP300. We focused our attention on representative lncRNA625-induced genes involved in enhancing cancer cell proliferation, invasion and migration. Use of qRT–PCR confirmed that lncRNA625 induced multiple PCGs, such as NEK6, TNC, KCTD12, NCOA4, CROT, HIST1H2BM, ASUN and CCNG1 after silencing of either EP300 or lncRNA625 (Figure 5h), consistent with the cDNA microarray results. Indeed, most of lncRNA625- and EP300-induced downstream PCGs evaluated by qRT–PCR are known positive regulators of cancer, including NEK6,^35, 36, 37, 38^ TNC,^39, 40^ CCNG1,^41^ HIST1H2BM,^42^ NCOA4^43^ and KCTD12.^44^ These results demonstrate the key role of EP300 in executing lncRNA625-induced transcriptional responses of genes, to promote cancer cell proliferation, invasion and migration.

### LncRNA625 constitutes a tumor signature for survival time in metastatic ESCC

**Figure 6:**
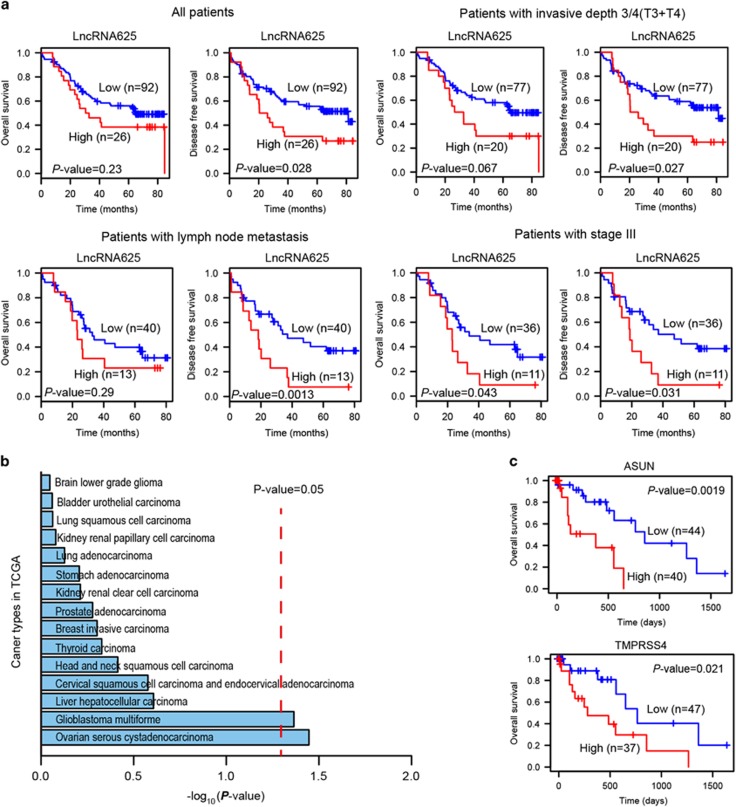
Kaplan–Meier curves of ESCC patients with either higher or lower expression of lncRNA625 and downstream target PCGs. (**a**) Kaplan–Meier survival curves of patients with ESCC classified into high- and low-risk groups based on their lncRNA625 signature. Expression level and survival information were obtained in 118 cases from 120 ESCC patient samples. For patients with invasive depth 3/4 (T3/T4), lymph node metastasis and stage III, a stratified analysis was done. (**b**) Kaplan–Meier survival based on the lncRNA625 signature for the cancer lncRNA profiles in TCGA. (**c**) Kaplan–Meier survival curves of ESCC patients classified into high- and low-risk groups based on two lncRNA625 downstream target PCG signatures. Expression level and patient information were obtained from TCGA. Red and blue indicates higher and lower expression, respectively.

The relationship between lncRNA625 expression and prognosis of ESCC patients was explored by Kaplan–Meier analysis and log-rank test. LncRNA625 levels were measured in an independent panel of 118 cases from 120 ESCC patients with extensive clinical follow-up. Dependent on the lncRNA625 signature, patients were divided into either a high-risk group (*n*=92) or a low-risk group (*n*=26). Patients with high lncRNA625 expression did not have significantly shorter overall survival (OS) than those with the low expression. For disease-free survival (DFS), patients with high lncRNA625 expression had significantly shorter survival time than those with low expression (median survival 23.2 months vs >80 months, *P*<0.028; Figure 6a). Thus, high lncRNA625 levels predicted poor prognosis.

To test whether lncRNA625 has prognostic value within clinical stages, a stratified analysis was performed on stage III ESCC patients to evaluate whether the lncRNA625 signature could predict survival of patients within the same clinical stage. A log-rank test showed that lncRNA625 could classify patients with stage III into high- and low-risk groups (Figure 6a). However, for patients with stage I/II, lncRNA625 level was not a significant predictor of prognosis (Supplementary Figure 7). Stratified analysis for patients with invasive depth 1/2 (T1/T2) and non-lymph node metastasis showed that high lncRNA625 expression did not confer shorter OS and DFS (Supplementary Figure 7). However, a stratified analysis for patients with either invasive depth 3/4 (T3/T4) or lymph node metastasis showed that lncRNA625 could be a significant predictor of subsequent metastasis and death. Taken together, lncRNA625 showed specific prognostic value for patients with metastatic ESCC (Figure 6a).

To assess whether the prognostic ability of the lncRNA625 signature is independent of other clinical or pathological factors of ESCC patients, multivariate Cox regression analysis with disease-free survival was performed using the Cox proportional hazards regression mode. Selected co-variables included age, gender, tumor size, TNM stage and lncRNA625. The results showed that survival prediction by lncRNA625 levels is independent of clinical and pathological factors for DFS of patients with ESCC (Supplementary Table 7).

To assess the prognostic ability of the lncRNA625 signature in other cancer, lncRNA profiles in TCGA were analyzed. We obtained lncRNA prognostic results of TCGA from the ‘My lncRNA' module in the TANRIC database (http://ibl.mdanderson.org/tanric/_design/basic/index.html). In the TANRIC database, a total of 15 cancer types are available for survival analysis. LncRNA625 showed moderate prognostic value in ovarian serous cystadenocarcinoma and glioblastoma multiforme patients, with a log-rank test *P*-value <0.05 (Figure 6b). For other cancer patients, lncRNA625 level was not a significant predictor of prognosis (Figure 6b). These suggested that lncRNA625 has the better prognostic ability in ESCC than other cancers.

As lncRNA625 showed prognostic value, downstream target PCGs of lncRNA625 might share a similar prognostic value. To test this, the mRNA profile of ESCC in TCGA was analyzed. Two lncRNA625 target PCGs (ASUN and TMPRSS4) showed prognostic value in ESCC patients (Figure 6c). TMPRSS4 promotes metastasis in preclinical models,^54^ and two independent studies have demonstrated the prognostic value of TMPRSS4 in breast cancer.^55, 56^ Our analysis determined that ESCC patients with high TMPRSS4 expression had significantly shorter survival time than those with low expression (*P*<0.021). Interestingly, ASUN has not been reported in cancer. However, ESCC patients with high ASUN expression had significantly shorter survival time than those with the low expression (*P*<0.0019), suggesting that ASUN regulation by lncRNA625 might be a new marker for poor prognosis of ESCC. Taken together, lncRNA625 and its downstream target PCGs have prognostic value for ESCC, especially in patients with metastasis.

## Discussion

We describe a comprehensive analysis of lncRNAs in 30 esophageal samples (15 ESCC tissue samples and 15 paired non-tumor tissues) by massive parallel next-generation sequencing platforms. Many lncRNAs whose expression patterns distinguish ESCC from normal tissue were identified, and are co-expressed with PCGs in the global lncRNA-PCG functional network. The functions of lncRNAs are closely associated with their abundance of transcripts and downstream target PCGs directly or indirectly regulated by them. An effective strategy to identify key lncRNAs is to develop network-based inference methods, which have successfully been used for disease PCG and ncRNA identification.^57, 58, 59^ Based on an ESCC-associated lncRNA-PCG functional network, we developed an unsupervised random walk method using dysregulated lncRNAs/PCGs as seeds for identification of functional lncRNAs. We focus more on identification of novel key lncRNAs by considering dysregulated lncRNAs/PCGs as a search source, and then performing a global search within a tumor-specific lncRNA-PCG functional network.

Based on URW-LPE, multiple known cancer and many novel potentially functional lncRNAs were effectively identified in ESCC. Some known functional lncRNAs, including HOTAIR, ANRIL, DLX6-AS1, AC130710.1.1, WT1-AS and LINC00261 displayed elevated URWScores compared with their FC values. As all candidate functional lncRNAs in ESCC cannot be validated, we focused on those lncRNAs that displayed highly differential expression and high rank. LncRNA625 was identified as a representative of a functional lncRNA in ESCC and was noted to be a novel regulator of cell invasion and migration in ESCC. Patients with high lncRNA625 expression had a significantly shorter DFS than those with low expression. LncRNA625 also displayed specific prognostic value for patients with metastatic ESCC. We further found that lncRNA625 interacts with EP300, and that multiple downstream genes of lncRNA625 are regulated by EP300. Some of these genes, including NEK6,^35, 36, 37, 38^ TNC,^39, 40^ CCNG1,^41^ HIST1H2BM,^42^ NCOA4^43^ and KCTD12^44^ are associated with cancer. These results demonstrate that lncRNA625 might have a role in ESCC by interacting with EP300 to simultaneously upregulate oncogenes and downregulate tumor suppressor genes associated with cell proliferation, invasion and migration in ESCC.

EP300 is a histone acetyltransferase that occupies tissue-specific transcriptional enhancers.^60^ EP300 was also reported to be able to bind lncRNAs such as lncRNA21.^61^ We found that EP300 regulates many downstream target genes of lncRNA625. This suggests that genes upregulated by lncRNA625 and EP300, such as TNC, NEK6, CCNG1, HIST1H2BM, NCOA4, NCOA4 and KCTD12, are highly likely to be induced by lncRNA625 via recruitment of EP300 to target gene promoters. Studies showed that motifs for transcription factors, such as ETS, FOX, AP1 and STAT, are enriched in EP300-bound regions, suggesting that complex regulatory mechanism of lncRNA625 and EP300 might be dependent on other transcription factors. We modulated the related EP300 network and showed that the downstream target gene network of lncRNA625 and EP300 likely very complex, causing numerous changes in global gene expression in ESCC patients (Supplementary Figure 8). This demonstrates that it is necessary to use a network-based global algorithm for identifying functional lncRNAs.

In summary, we used transcriptome sequencing technology to profile the transcriptomes of both ESCC and non-tumor tissues of 15 Chinese patients. Many novel potentially functional lncRNAs with high statistical significance were effectively identified by our URW-LPE method. Of these, lncRNA625 was found to be a novel biomarker for prognosis of patients with ESCC and to regulate cell proliferation, invasion and migration through interacting with EP300. Our findings support an important role for lncRNAs in ESCC and suggest that functions of these lncRNAs may help to ‘drive' cancer initiation and progression. Our data provide an important resource for future studies of key lncRNAs in ESCC and establish the utility of integrative bioinformatic analyses of RNA-seq to identify functional cancer-associated lncRNAs.

## Materials and methods

### Sample collection and preparation

All patients were from the Chaoshan District of Guangdong Province, one of the high prevalence ESCC areas in China.^62^ Samples were collected from the Department of Oncological Surgery of the Central Hospital of Shantou City, China. Documented informed consent was obtained through the institutional review board during 2007–2013. Tumor and paired non-tumor tissues were collected from each patient who underwent surgical resection; none of the patients were treated with chemotherapy or radiotherapy before operation. After being examined by a pathologist, tissues were immediately frozen in liquid nitrogen and stored at −80 °C. Partial tissues were used for haematoxylin and eosin staining to confirm the diagnosis and analysis of pathological grade, metastasis and tumor cell content. All tumor samples contained more than 80% free of necrosis. Cases were classified according to the tumor-node-metastasis (TNM) classification of the International Union against Cancer, 7th edition. Evaluation of tumor differentiation was based on histological criteria of the guidelines of the WHO Pathological Classification of Tumors. This study was approved by the Ethics Committee of the Central Hospital of Shantou City.

### RNA-seq library preparation

Total RNA was isolated using an RNeasy mini kit (Qiagen, Hilden, Germany) and analyzed on 1% formaldehyde-denatured agarose gels to ensure no degradation occurred. Paired-end libraries were synthesized by using the TruSeq RNA Sample Preparation Kit (Illumina, USA) following the TruSeq RNA Sample Preparation Guide. Briefly, poly-A containing mRNA was purified using poly-T oligo-attached magnetic beads. Following purification, the mRNA was fragmented into small pieces by using divalent cations at 94 °C for 8 min. The cleaved RNA fragments were copied into first strand cDNA using reverse transcriptase and random primers, followed by second strand cDNA synthesis using DNA polymerase I and RNase H. cDNA fragments underwent an end repair process, the addition of a single ‘A' base, and then ligation of the adapters. Products were purified and enriched by PCR to create the final cDNA library. Purified libraries were quantified using a Qubit 2.0 Fluorometer (Life Technologies, Carlsbad, CA, USA), and validated with an Agilent 2100 bioanalyzer (Agilent Technologies, Santa Clara, CA, USA) to confirm the insert size and calculate the mole concentration. A cluster was generated by cBot, with the library diluted to 10 pM, and then inserts were sequenced on the Illumina HiSeq 2500 (Illumina, San Diego, CA, USA). Library construction and sequencing was performed at the Shanghai Biotechnology Corporation. Transcriptome sequencing data are available publicly at the Sequence Read Archive (http://www.ncbi.nlm.nih.gov/sra/) under accession number SRP064894.

### Identification of esophageal cancer-related lncRNAs and coding genes

Expression profiles of 15 paired ESCC and non-tumor samples were extracted by using Tophat (v2.0.6, Tophat, Washington, MD, USA) and easyRNAseq version 1.6.0 (Heidelberg, Germany), in which the lncRNAs and PCGs were included. Then, we used DESeq version 1.14.0 (Heidelberg, Germany) to identify the differentially expressed lncRNAs and coding genes based on the count number expression profile, and we used the fold change method to estimate the differential significance of lncRNAs and coding genes based on the RPKM expression profile. In this study, esophageal cancer-related differentially expressed lncRNAs/coding genes are defined as the lncRNAs/coding genes with a fold change >2 or <½, and a DESeq FDR value <0.25.

### Construction of the ESCC-associated lncRNA-PCG functional network

The ESCC-associated lncRNA-PCG functional network belongs to an extended lncRNA-PCG co-expression network. First, an ESCC-specific lncRNA-PCG co-expression network was constructed based on differentially expressed lncRNAs and PCGs. We calculated the Pearson correlation coefficient between the differentially expressed lncRNAs and PCGs. *P*-value of Pearson correlation coefficient of each lncRNA-PCG pair was evaluated by Fisher's asymptotic test, which is implemented in the WGCNA R package of version 1.34, and Bonferroni correction method was used to control the false discovery rate. Second, protein interaction data was collected from the HPRD database and the lncRNA-protein interaction data from the NPInter v2.0 database. The protein interaction data in HPRD (http://www.hprd.org/) has been manually extracted from the literature by expert biologists. The NPInter database (http://www.bioinfo.org/NPInter) contains experimentally verified interactions between non-coding RNAs and proteins. PCG-PCG interactions were extracted from the co-expression data and HPRD database. Specifically, if the corrected co-expression *P*-value of two differential PCGs was less than 1.0e^−7^ or they had direct interaction in the HPRD network, the PCG interaction pair was extracted. Similarly, we extracted the lncRNA-PCG interaction pairs if the corrected co-expression *P*-value of the differential lncRNA and PCG was less than 1.0e^−7^ or they had direct interaction in the NPInter network. Finally, the ESCC-associated lncRNA-PCG functional network was constructed by combining the extracted lncRNA-PCG and PCG-PCG interaction pairs.

### URW-LPE

We developed an integrative bioinformatics method, denoted URW-LPE, for identification of functional lncRNAs in ESCC (URW-LPE is available at https://github.com/LICLAB/URW-LPE). In brief, a random walk was run with each dysregulated lncRNA/PCG as a seed and an extended co-expression relation as an edge. First, an extended lncRNA-PCG co-expression network was constructed based on differentially expressed lncRNAs and PCGs in ESCC. Second, we ran the random walk for the network, considering the fold change (FC) values of each node on the network as the initial probability vector. As a result, each lncRNA in the network would be given an URWScore value. The higher URWScore value of lncRNAs represents more important functions in ESCC. Finally, the FDR-corrected *P*-value for each lncRNA in the network was calculated through comparison of the real URWScore of each lncRNA and that of the background distribution. This method can identify lncRNAs based on global network information, which usually represents indirect target relations. Compared with other methods, we focused more on considering dysregulated lncRNAs/PCGs as a search source, and performing global searching within the ESCC-specific lncRNA-PCG co-expression network.

Specifically, we used a variant of the random walk with a restart of probability r in each iterative step, and the fold change values of each node on the network were considered as the initial probability vector. The formula of the random walk can be represented as follows:


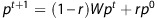


where W is the adjacency matrix of the lncRNA-PCG co-expression network, which has been row-normalized, *p*^t^ is a vector that the element of which represents the probability of the corresponding lncRNA and PCG nodes at step *t*, *p*^0^ is the initial probability vector (used as the setting seed). In previous methods, *p*^0^ was constructed according to known disease lncRNAs (or genes).^22^ However, for many diseases, such as ESCC, only a few lncRNAs are available to be used as seeds, which causes decreased predictive power for a single disease. We thus used each dysregulated lncRNA/PCG as a seed, but with a different importance score according to the fold change (FC) value. Specifically, *p*^0^ was constructed according to the fold change value of nodes. For a given node*i* (lncRNA or PCG), we define *p*^0^ as the initial probability of node *i*, which is computed as follows:


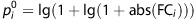


where FC*_i_* is the log_2_-transformed fold change value of node *i*. Finally, each differential lncRNA was given an URWScore value. The higher URWScore value of lncRNAs, the more important functions in ESCC.

To identify the statistically significant lncRNAs, *P*-values for each lncRNA in the network were calculated through comparison of the real URWScore of each lncRNA and that of the background distribution. For lncRNAs in the network, we randomly generated FC values of each lncRNA from a normal distribution with the same mean and s.d. as the real FC values of lncRNAs. For PCGs in the network, FC values of PCGs in the network were shuffled. The URWScore of each lncRNA was re-computed using the above random walk method. A total of 1 230 000 (615 × 2000) URWScores of lncRNAs was generated and used as the background distribution. The *P*-value is the fraction of the number of lncRNAs, which is larger than that in the real URWScore. FDR-corrected *P*-values for each lncRNA in the network were calculated using the Benjamini–Hochberg FDR method.

### Cell culture

The SHEEC human ESCC cell line and SHEE immortalized human esophageal cell line were established in our laboratory^63^ and maintained in DMEM/F12 (1:1) medium containing 10% newborn bovine serum. The KYSE150, KYSE450, KYSE510 human esophageal cancer cell lines were kindly provided by Dr. Ming-Zhou Guo (Chinese PLA General Hospital, Beijing, China) and cultured in RPMI-1640 medium supplemented with 10% fetal bovine serum. KYSE150, KYSE450 and KYSE510 cells were derived from human esophageal cancer cells. The HEK293T human embryonic kidney cell lines and NIH3T3 mouse embryonic fibroblast cells were kindly provided by Professor Dong Xie (The Institute for Nutritional Sciences, Chinese Academy of Sciences, China). HEK293T cells were derived from human embryonic kidney cells and NIH3T3 was derived from mouse embryonic fibroblast cells. All the cell lines were authenticated by STR profiling and tested for mycoplasma contamination and grown at 37 °C under a humidified atmosphere of 5% CO_2_.

### Human lncRNA625 cloning and expression plasmid construction

RNA was extracted and contaminating genomic DNA was removed by DNase I. The cDNA synthesis was performed using a cDNA synthesis kit (6210A, Takara, Dalian, China), and the reverse primer was used as the gene-specific primer during the cDNA synthesis. The full-length 577-bp lncRNA625 transcript (chromosome 15:62, 682, 916-62, 690, 448, reverse strand) was amplified by PCR using Pfu DNA polymerase (Transgene, Beijing, China) with the forward primer containing a Hind III site and the reverse primer containing a Not I site. The PCR product was cloned into the pcDNA3.1 eukaryotic expression vector (Life Technologies) and confirmed by sequencing.

### Transfection of siRNAs and plasmids

KYSE150 or KYSE510 cells were inoculated in a 12-well plate, containing antibiotic-free medium, for 24 h to achieve the desired density of 30-50% confluence prior to transfection. The siRNA oligos were synthesized by GenePharma (Shanghai, China). For small interfering RNA, 1.6 μg siRNA oligos and 8 μl transfection reagent (Roche, Indianapolis, IN, USA) were mixed in Opti-MEM culture medium (Life Technologies), and the mixture of siRNA and transfection reagent was added to each well. The procedure for transfection was performed according to the protocol provided by the transfection reagent supplier. For the lncRNA625 construct, 70-90% confluence was achieved at the time of plasmid transfection and 1.6 μg lncRNA625 expression plasmid and 4.8 μl Lipofectamine 3000 transfection reagent (Life technologies) were used per well. The sequence of siRNA oligos used in this study was as follows: lncRNA625: 5′-GACCACCAUCAAGGGAUAAdtdt-3′ (sense); 5′-UUAUCCCUUGAUGGUGGUCdtdt-3′ (antisense). EP300:5′-ACAGCUGUCAGAAUUGCUGdtdt-3′ (sense); 5′-CAGCAAUUCUGACAGCUGUdtdt-3′ (antisense). Scrambled RNA (negative control):5′-UUCUCCGAACGUGUCACGUdtdt-3′ (sense); 5′-ACGUGACACGUUCGGAGAAdtdt-3′ (antisense).

### Construction of stable shlncRNA625-expressing cells

A shlncRNA625 lentiviral vector was constructed by Hanbio (Shanghai, China) according to the above siRNA oligo sequence against lncRNA625. KYSE150 or KYSE510 cells were inoculated in a 24-well plate, containing normal culture medium, at 2.5x10^4^ cells per well. The next day, 5 μl lentiviral solution containing shlncRNA625 vector was mixed with 250 μl normal culture medium with polybrene (Sigma-Aldrich, St Louis, MO, USA) at a final concentration of 6 μg/ml, and then the mixture was used to infect cells after the removal of the previous culture medium. After 4 h, an additional 250 μl normal medium with polybrene was added to each well. The virus-containing culture medium was removed at 24 h post-transfection, fresh medium was added for an additional 24 h. and then cells were refed with culture medium containing puromycin (final concentration 500 ng/ml). The level of lncRNA625 on the fifth or sixth passage was quantified by real-time RT–PCR in order to screen multiple cell clones for lncRNA625 knockdown. Control cells were obtained by infection with virus encoding a scrambled short hairpin RNA.

### Invasion, migration and colony formation assays

KYSE150 or KYSE510 ESCC cells were subjected to lncRNA625 knockdown or upregulation by either RNA interference or overexpression of lncRNA625, respectively. At 24 h post transfection, cells were starved for 12 h with serum-free culture medium and then cell invasion, migration and colony formation assays were performed according to previously described methods.^64, 65^ Briefly, 5 × 10^4^ cell were plated in medium without serum in the upper well of a transwell chamber with (for cell invasion) or without (for cell migration) a Matrigel-coated membrane (24-well insert; pore size, 8 μm; BD Biosciences, Franklin Lakes, NJ, USA), and with the lower chamber containing medium supplemented with 10% serum. The cells were incubated for 48 h and the cells in the top transwell chamber that did not invade or migrate through the pores were removed with a cotton swab. Cells that invaded or migrated through the pores were fixed and stained with haematoxylin solution, and counted. Stable cell lines expressing either shlncRNA625 or control lentiviral vector were used in identical cell invasion and migration assays. For colony formation assays, 1x10^3^ cells/per well were inoculated in each well, of a six-well plate, containing medium+10% fetal bovine serum. Colonies were stained with haematoxylin solution and observed after incubation for 15 days.

### Tumor xenografts

Animal experiments were approved under the guidelines of the Animal Policy and Welfare Committee of Shantou University Medical College. Six-week-old female BALB/c nude (nu/nu; *n*=9) mice purchased from Beijing Weitonglihua Company in China were anesthetized with an isoflurane/propylene glycol mixture and KYSE150-shlncRNA625 or shscramble stable cells were subcutaneously injected into the right flanks (1.0 × 10^6^ cells/mouse). After one week, the tumor volume was measured every two days according to the formula V=ab^2^/2 (‘a' means the length of tumor tissue and ‘b' means the width of tumor tissue). All mice were euthanized using the inhalation of CO_2_ at three weeks and tumors were collected and weighed.

### Reverse transcription (RT) and real-time PCR

Total RNA was extracted using TRIzol (15596-018, Life Technologies) and purified with a PureLinkTM RNA Mini Kit (12183018A, Life Technologies) according to the manufacturer's protocol. The purity and concentration of RNA were determined by OD260/280 using a NanoDrop ND-2000 spectrophotometer. cDNA synthesis was made by reverse transcription, and real-time PCR was performed by using a SYBR Premix Ex Taq kit (DRR037A, DRR081A; Takara). Briefly, reverse transcription was performed according to the following conditions: 37 °C, 15 min; 85 °C, 5 s. Real-time PCR was performed using an ABI 7500 real-time PCR system (Life Technologies) according to the following conditions: 95 °C, 30 s; 95 °C, 5 s; 60 °C, 34 s. Relative quantification of mRNA expression was calculated by the 2-ΔCt method.

### LncRNA625 detection in cytoplasmic and nuclear extracts

Cytoplasmic and nuclear extracts were prepared according to an online protocol (http://www.lifetechnologies.com/cn/zh/home/references/protocols/cell-and-tissue-analysis/elisa-protocol/elisa-sample-preparation-protocols/nuclear-extraction-method-.html). The cytoplasmic extract was mixed with chloroform and centrifuged at 12400 g and 4 °C after vortexing. The upper layer was collected, and RNA was isolated with an RNA extraction kit (DP419, Tiangen Biotech, Beijing, China). RNA from the nuclear extract was extracted by using TRIzol (Life Technologies), and lncRNA625 was subjected to reverse transcription and real-time PCR. Cytoplasmic and nuclear extracts from KYSE510 cells were prepared, and U6 snRNA was used as the nuclear control and GAPDH mRNA was used as the cytoplasmic control transcript.^66^

### Fluorescence *in situ* hybridization

The PCR product used as the template for lncRNA625 fluorescence *in situ* hybridization (FISH) probe synthesis was amplified according to the appropriate reference.^67^ Briefly, we designed the forward primer with T7 promoter sequence for sense probe synthesis and the reverse primer with T7 promoter sequence for antisense probe and then the pcDNA3.1-lncRNA625 vector was used as the template to amplify the PCR product for FISH probe synthesis. The primers for sense probe synthesis were as following: Forward:5′-GATCACTAATACGACTCACTATAGGGAGAGACCACCATCAAGGGATAAAAT-3′ Reverse:5′-GGCTAATAAACAGGGTCTTCAGGT-3′ the primers for antisense probe synthesis were 5′-AGAGACCACCATCAAGGGATAAAAT-3′(Forward), 5′-GATCACTAATACGACTCACTATAGGGGGCTAATAAACAGGGTCTTCAGGT-3′(Reverse). FISH probe synthesis was performed according to the following condition: 1 μg PCR product, 2 μl T7 RNA polymerase, 2 μl 10 × buffer, 2 μl biotin RNA labeling mix, 0.5 μl RNase inhibitor and water was added to total 20 μl volume. The mix was incubated at 37 °C for 2 h and the probes were diluted with deionized formamide at 1:5.

FISH assays were performed using the method the reference provided with minor modification.^68^ Three-μm tissue sections were subjected to deparaffinization and dehydration. After 0.1 N HCl treatment, tissue sections were heated in citrate buffer (pH 6.0) for 20 sec every 2 min in a microwave for a total of 12 min and were subsequently digested using proteinase K at 20 μg/ml for 20 min at 37 °C, and then the tissues were fixed in 4% paraformaldehyde for 10 min at room temperature. Sections were prehybridized for 2 h at 37 °C in hybridization buffer: 1 μl DTT, 50 × Denhardt's solution (Suolaibao, Beijing, China), 1 μl salmon sperm ssDNA (Life Technology), 2 μl 20 × SSC, 10 μl deionized formamide, 2 μl 50% sodium dextran sulphate). The FISH probes, sense or antisense probe for lncRNA625, were added to hybridization buffer. After denaturation at 80 °C for 10 min, the probes were incubated with tissue sections overnight at 48 °C and then sections were washed in 2 × SSC containing 50% formamide and 2 × SSC twice at 37 °C for 15 min. Cy3-conjugated streptavidin (ThermoFisher Scientific, Hudson, NH, USA) was subsequently incubated with the slides at room temperature for 1 h, and then fluorescent signals were observed with a fluorescence microscope Axio Imager A2 (Zeiss, Bochum, Germany) after DAPI staining. Serial slides were stained with haematoxylin and eosin to confirm tumor histomorphology.

### RNA immunoprecipitation

RNA immunoprecipitation was performed according to a modified method of Yoon*et al.*^66^ Briefly, KYSE150 and KYSE510 cells were lysed in lysis buffer (20 mm Tris-HCl, pH 7.5, 100 mm KCl, 5 mm MgCl_2_, 0.5% NP-40, protease inhibitors, 200 U/ml RNase inhibitor) for 15 min on ice, and then supernatants were collected by centrifugation at 10000 g for 15 min at 4°C. To completely lyse the nuclear membrane, the pellet was vortexed at 4 °C, and then cell debris was removed by centrifugation at 16 000 g for 10 min. All supernatants were combined and 10% of the combined supernatant was used as input and equivalent quantities of supernatants were incubated overnight at 4 °C with magnetic beads coated with protein G and antibodies, recognizing either rabbit anti-EP300 (sc-585, Santa Cruz Biotechnology, Santa Cruz, CA, USA), rabbit anti-SP1 (ab13370, Abcam, USA), or rabbit normal control IgG (sc-2027, Santa Cruz Biotechnology). The next day, the magnetic bead complexes were subjected to extreme washing with lysis buffer and RNA was extracted with TRIzol (Life Technologies). RNA from 10% input was also extracted by TRIzol. Contaminating genomic DNA in the extracted RNA was removed by DNase I before reverse transcription, and real-time RT-PCR was subsequently performed using a PrimeScript RT reagent kit with gDNA Eraser. Real-time PCR for lncRNA625 was performed with a SYBR Premix Ex Taq kit (RR047A, DRR081A; Takara).

### cDNA microarray assay

Stably transfected KYSE150 cells, transfected with shlncRNA625 or shscramble, were collected and lysed in TRIzol (Life Technologies), and EP300 from human KYSE150 cells was subjected to knockdown by siRNA interference. Microarray experiments were performed by following the Affymetrix protocol at the Shanghai Biotechnology Corporation. Total RNA was isolated and purified by using an RNeasy Total RNA Isolation Kit and RNeasy Mini Kit (Qiagen, Hilden, Germany) according to the manufacturer's instructions. Total RNA was checked for a RIN number to inspect RNA integration by an Agilent Bioanalyzer 2100 (Agilent technologies). RNA samples from each group were then used to generate biotinylated cRNA targets for the Affymetrix GeneChip Human Transcriptome Array 2.0. The biotinylated cRNA targets were then hybridized with the microarray. After hybridization, arrays were stained in a Fluidics Station 450 and scanned on an Affymetrix Scanner 3000. Fluorescent signal intensities for all spots on the arrays were analyzed using a Gene Chip Operating System (Affymetrix, Cleveland, OH, USA). Ratios were calculated between either shlncRNA625 and shscramble, or siEP300 and shscramble. Genes with a fold change of at least two were selected for further analysis. The selected genes were grouped in functional categories based on the Gene Ontology database (GO: http://www.geneontology.org/). cDNA microarray data are available publicly at Gene Expression Omnibus (http://www.ncbi.nlm.nih.gov/geo/) under accession number GSE74707 and GSE74742.

### Statistical analysis

Statistical analyses were performed using SPSS 19.0 (IBM, Chicago, IL, USA) or R 3.1.2 (Auckland, New Zealand) for Windows. Comparisons of the relative expression of lncRNAs, between paired tumor and non-tumor tissues, were performed using a paired *t*-test and the above statistical method was also used for the statistical analysis of tumor volume and weight from the tumor tissues in mice xenograft. The comparisons between shscramble and siRNA cells were performed using the Mann–Whitney *U*-test. Overall survival time or disease-free survival time was calculated by the Kaplan–Meier method and analyzed by the log-rank test. Overall survival was measured from the date of surgery to death from any cause, and disease-free survival was measured from the date of surgery to disease progression or relapse. The optimal cutpoint for lncRNA625 expression was assessed by the X-tile program.^69^ Univariate and multivariate analyses were based on the Cox proportional hazards regression model. A two-tailed *P*-value <0.05 was considered to have statistical significance.

### Transcript profiling

Transcriptome sequencing data are available publicly at the Sequence Read Archive (http://www.ncbi.nlm.nih.gov/sra/) under accession number SRP064894. cDNA microarray data are available publicly at Gene Expression Omnibus (http://www.ncbi.nlm.nih.gov/geo/) under accession number GSE74707 and GSE74742.

## Figures and Tables

**Table 1:** Statistically significant functional lncRNAs predicted by URW-LPE

*LncRNA ID*	*LncRNA names*	*URW Score*	*FDR*	*Rank (URW Score)*	*Rank (FC)*
ENSG00000228630	HOTAIR^a^	0.51	0.025	35	93
ENSG00000231764	DLX6-AS1^a^	0.52	0.023	31	199
ENSG00000236289	AC130710.1.1^a^	0.54	0.017	15	78
ENSG00000183242	WT1-AS^a^	0.52	0.022	28	1
ENSG00000259974	LINC00261^a^	0.47	0.055	73	107
ENSG00000240498	ANRIL^a^	0.37	0.22	241	349
ENSG00000259756	RP11-625H11.2.1^b^	0.59	0.0077	3	1
ENSG00000233532	LINC00460^b^	0.50	0.032	43	130
ENSG00000230838	AC093850.2.1^b^	0.48	0.044	59	103

Abbreviations: lncRNA, long non-coding RNA; qRT–PCR, quantitative reverse transcriptase–PCR; URW, Unsupervised Random Walk method.

a Literature-evidenced functional lncRNAs in cancer.

b Novel functional lncRNAs, of which expression levels were confirmed by qRT–PCR in an additional 120 paired ESCC patient samples.
