# Supplementary material for: Integrative analyses of transcriptome sequencing identify novel functional lncRNAs in esophageal squamous cell carcinoma
Source: Oncogenesis. 2017 Feb 13;6(2):e297–. doi: 10.1038/oncsis.2017.1 (PMC5337622; doi:10.1038/oncsis.2017.1)
Supplement: Supplementary legends [file oncsis20171x1.docx]

**SUPPLEMENTARY FIGURE AND TABLE LEGENDS**

**Supplementary Figure 1. Heat maps of PCG expression levels.** Tree diagram exhibits a 2-branch partition with the 15 ESCC tumor samples clustered together and well separated from their matched non-tumor controls.

**Supplementary Figure 2. Expression of ANRIL and SOX2OT lncRNAs at the exon level.**

**Supplementary Figure 3. Properties of the lncRNA-PCG co-expression network.** (A) Degree distribution of the network. (B) Path length distribution of the network. Plot of topology coefficients and degree of neighbors. (D) Plot of average clustering coefficient and degree of neighbors.

**Supplementary Figure 4. LncRNA625 knockdown decreases the capacity of cell invasion and migration.** Invasion and migration capacity of KYSE150 and KYSE510 cells using transwell chambers were determined at 48 h post siRNA against lncRNA625 transfection. Levels of lncRNA625 were simultaneously detected by real-time RT-PCR.

**Supplementary Figure 5.** Promoter regions of a key lncRNA625-regulated PCG (NEK6) in the UCSC genome browser.

**Supplementary Figure 6. Predicted interaction of lncRNA625 and EP300 protein using the catRAPID predictor of protein-RNA binding.** (A) LncRNA625 interaction profile that is an average of interaction propensity over the EP300 protein fragment. (B) Interaction matrix that shows interaction propensity of individual the EP300 protein and lncRNA625 fragments.

**Supplementary Figure 7. Stratified analysis for patients with invasive depth 1/2 (T1/T2), non-lymph node metastasis and stage I/II.** Kaplan–Meier survival curves of patients with ESCC classified into high- and low-risk groups based on the lncRNA625 signature. Expression level and survival information were obtained from 118 cases of 120 ESCC patient samples. Red indicates higher expression and blue indicates lower expression.

**Supplementary Figure 8. EP300 regulatory network that displays the interaction of EP300 with some transcription factors to regulate lncRNA625 downstream target PCGs.**

**Supplementary Table 1. Clinical characteristics of patients with ESCC for RNA-seq analysis.**

**Supplementary Table 2. RNA sequencing information of 15 paired ESCC and non-tumor tissues**

**Supplementary Table 3. Statistically significant functional lncRNAs identified by URW-LPE.**

**Supplementary Table 4. Clinicopathological characteristics of patients with ESCC for qRT-PCR.**

**Supplementary Table 5. Primer sequences for PCR and real-time RT-PCR.**

**Supplementary Table 6. EP300 ChIP-seq data information of cell lines obtained from UCSC databases.**

**Supplementary Table 7. Univariate and multivariate analysis of factors associated with disease-free survival.**
