# Supplementary figures and images for: Integrative analyses of transcriptome sequencing identify novel functional lncRNAs in esophageal squamous cell carcinoma
Source: Oncogenesis. 2017 Feb 13;6(2):e297–. doi: 10.1038/oncsis.2017.1 (PMC5337622; doi:10.1038/oncsis.2017.1)

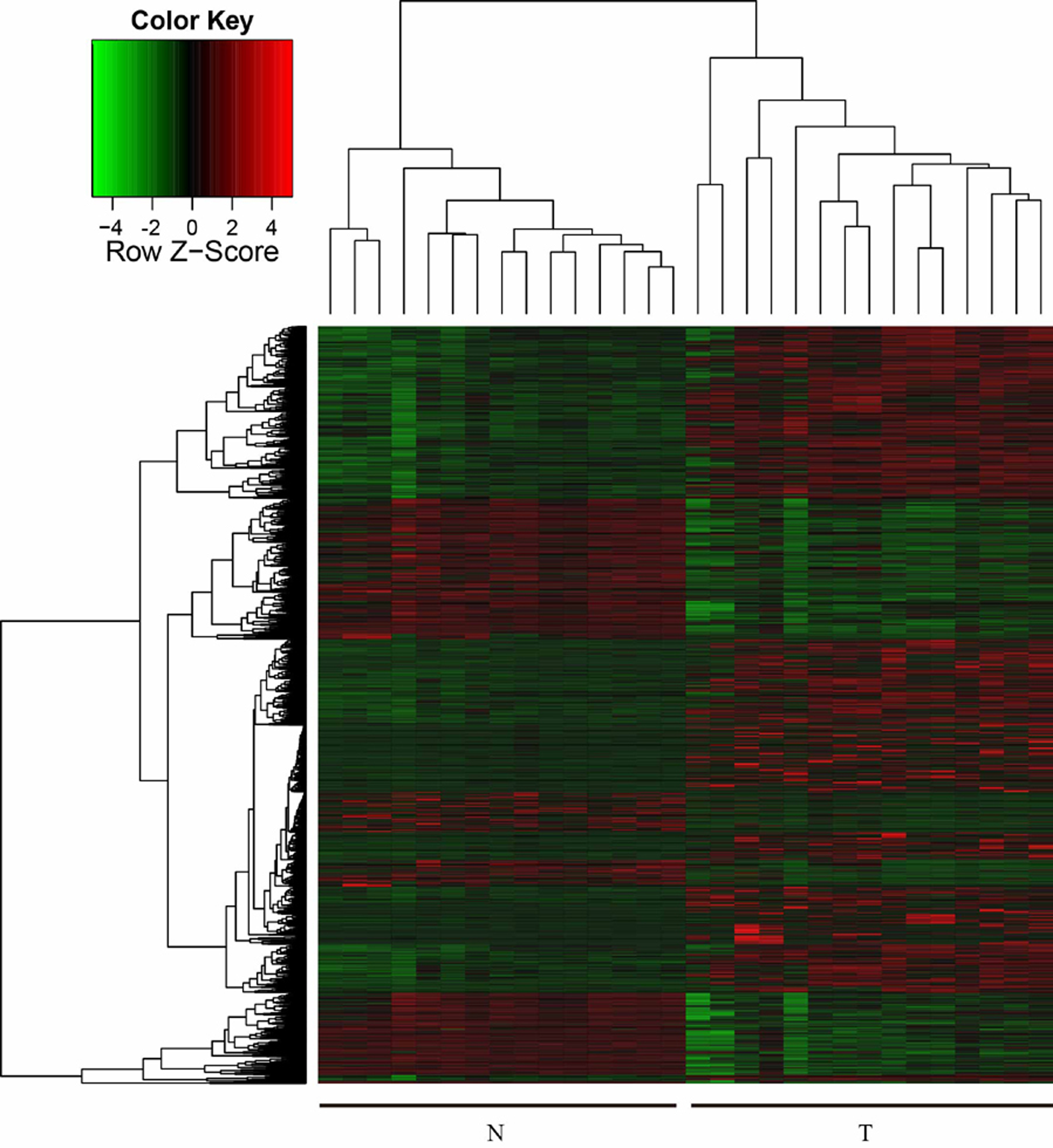

Supplement: Supplementary Figure 1 [file oncsis20171x2.tif]

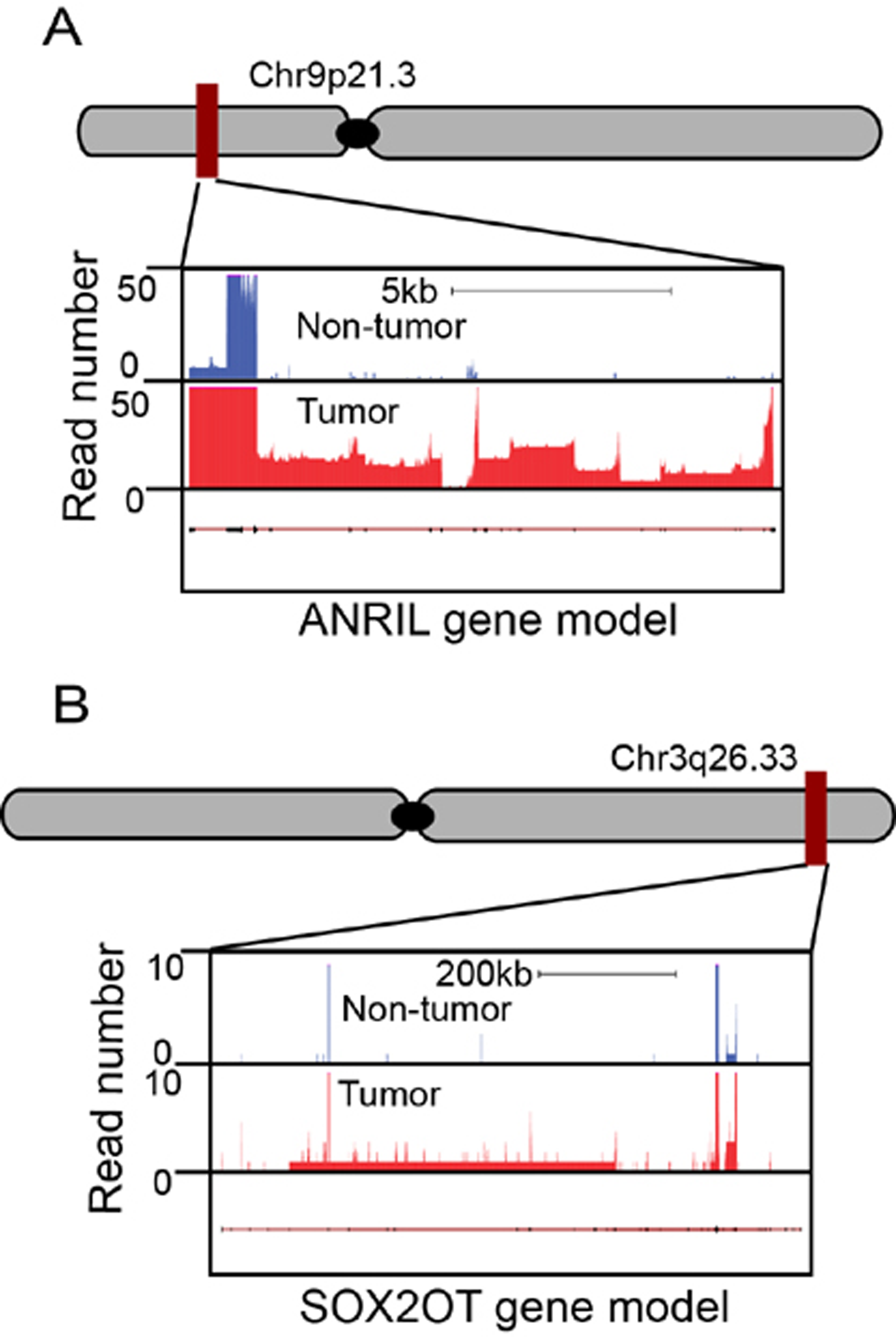

Supplement: Supplementary Figure 2 [file oncsis20171x3.tif]

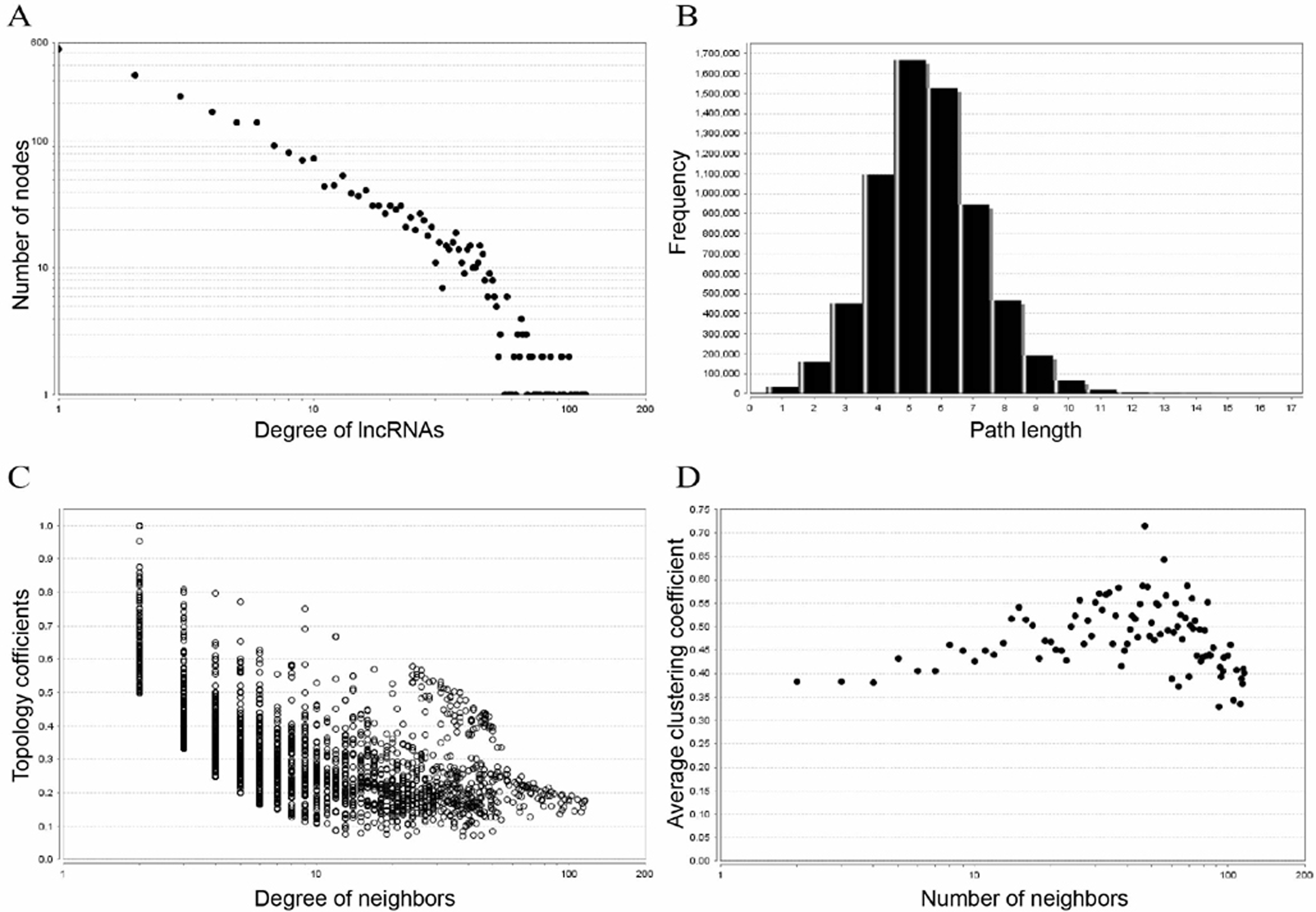

Supplement: Supplementary Figure 3 [file oncsis20171x4.tif]

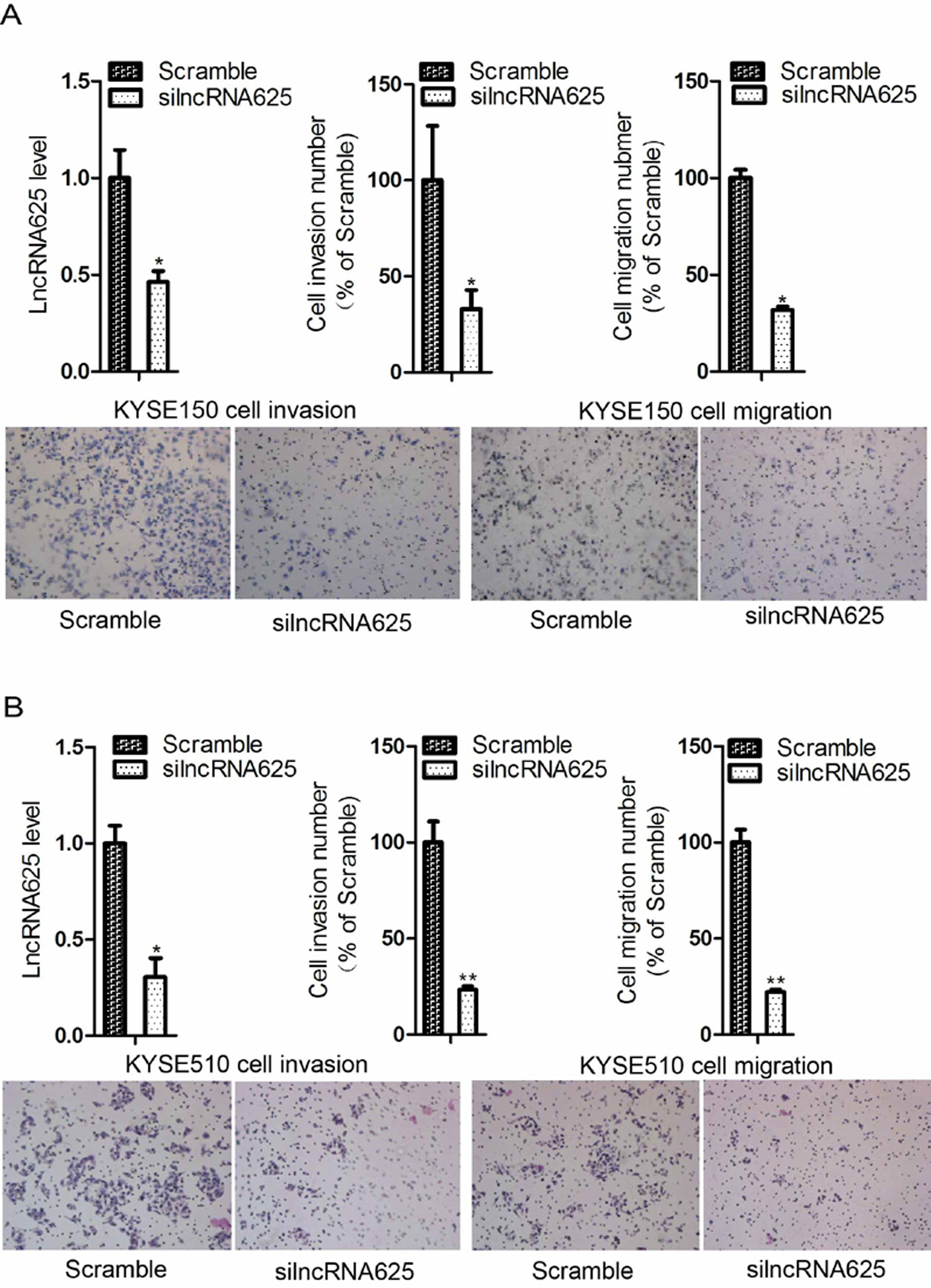

Supplement: Supplementary Figure 4 [file oncsis20171x5.tif]

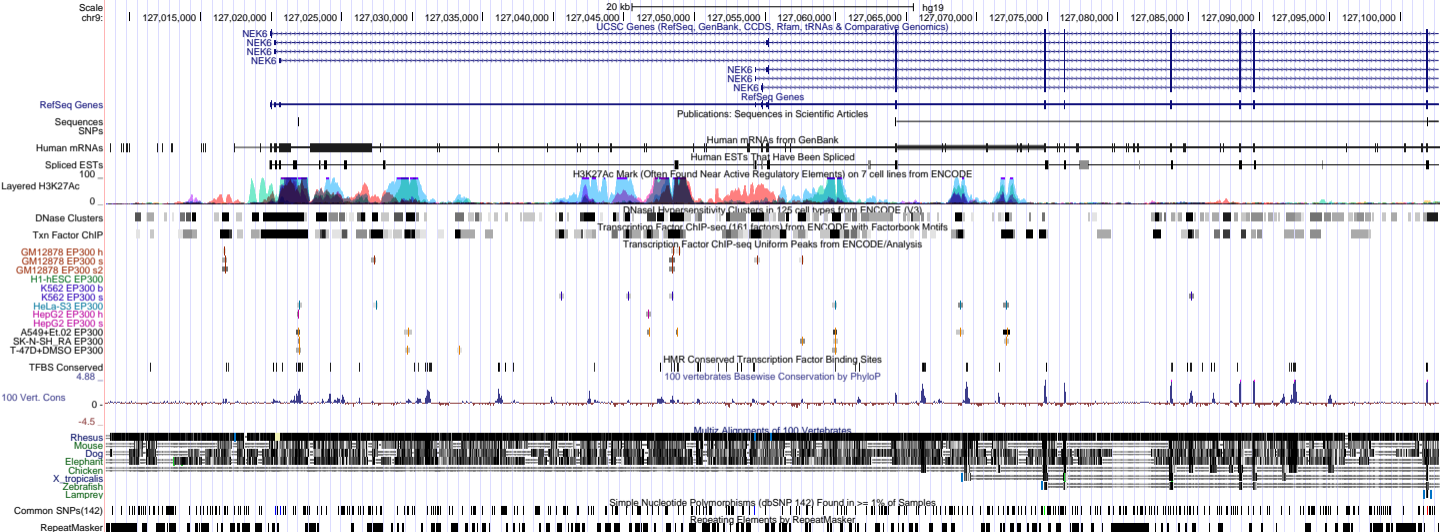

Supplement: Supplementary Figure 5 [file oncsis20171x6.pdf]

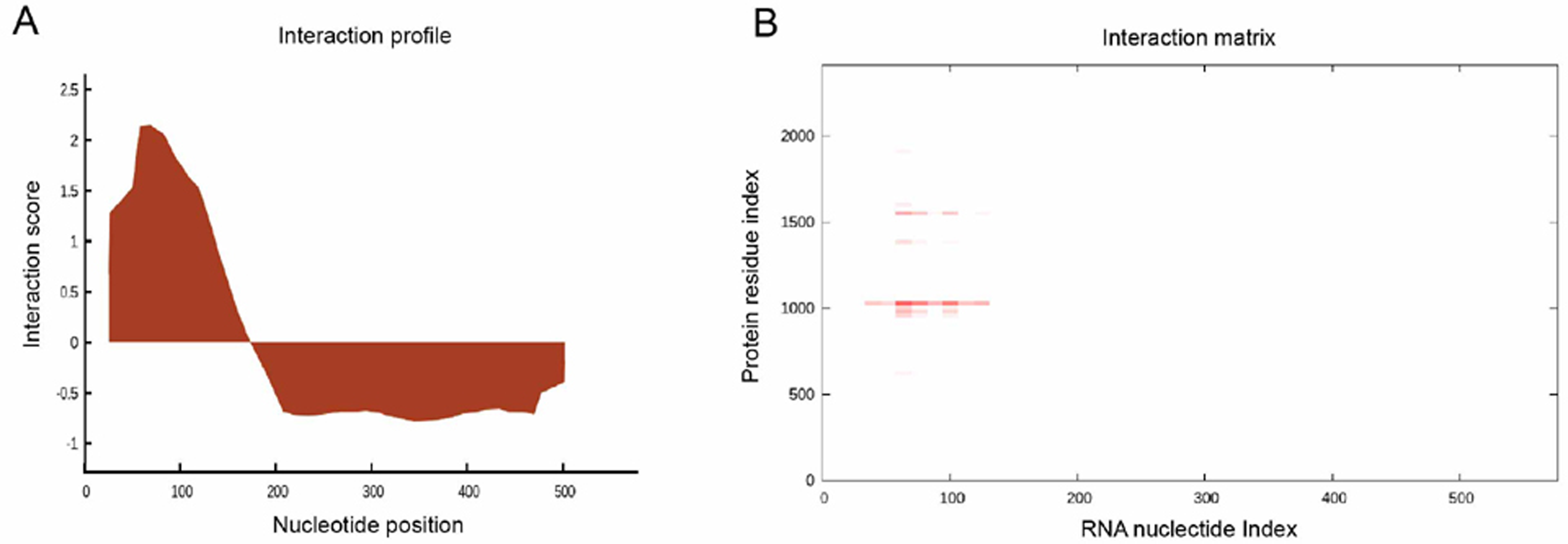

Supplement: Supplementary Figure 6 [file oncsis20171x7.tif]

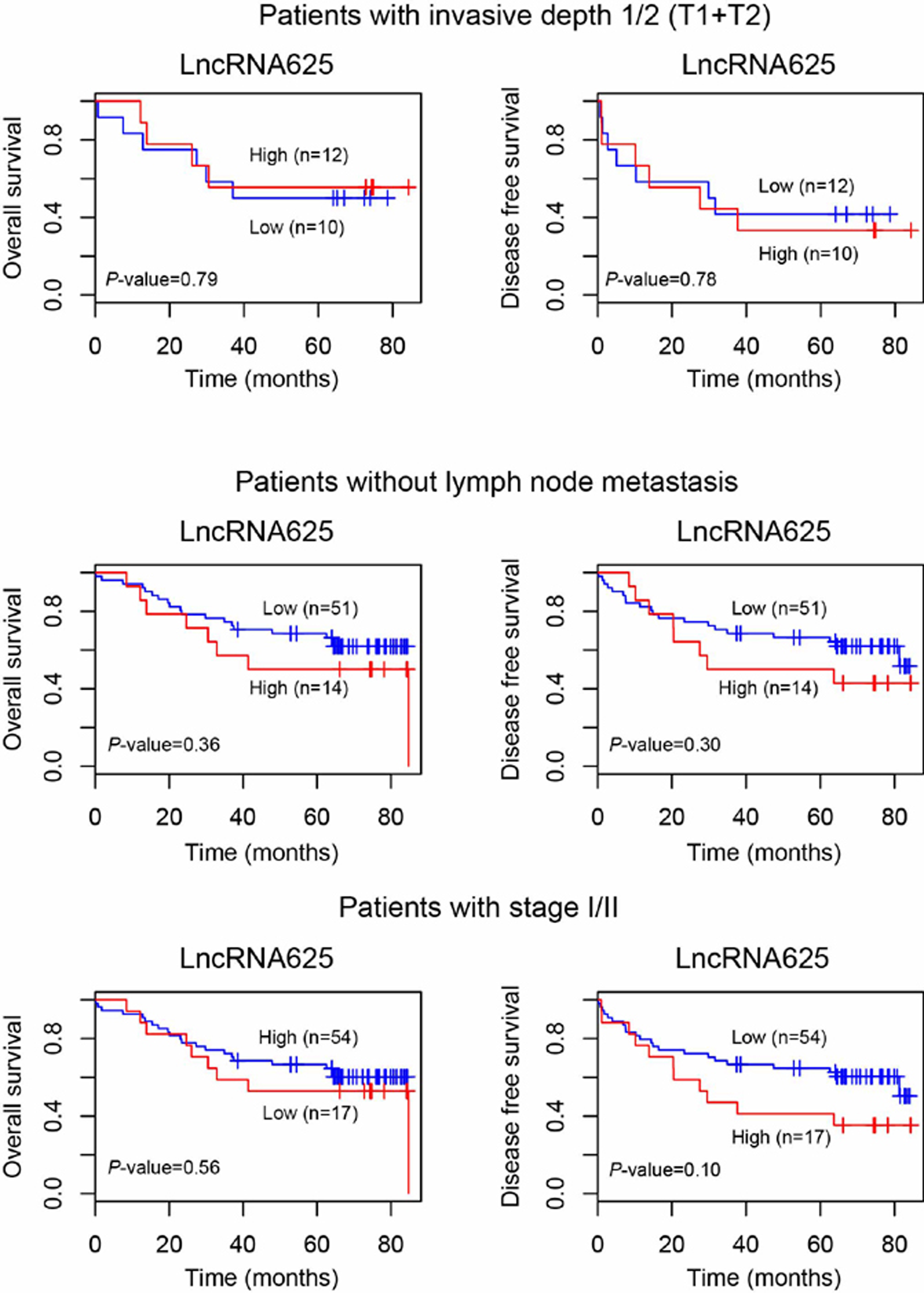

Supplement: Supplementary Figure 7 [file oncsis20171x8.tif]

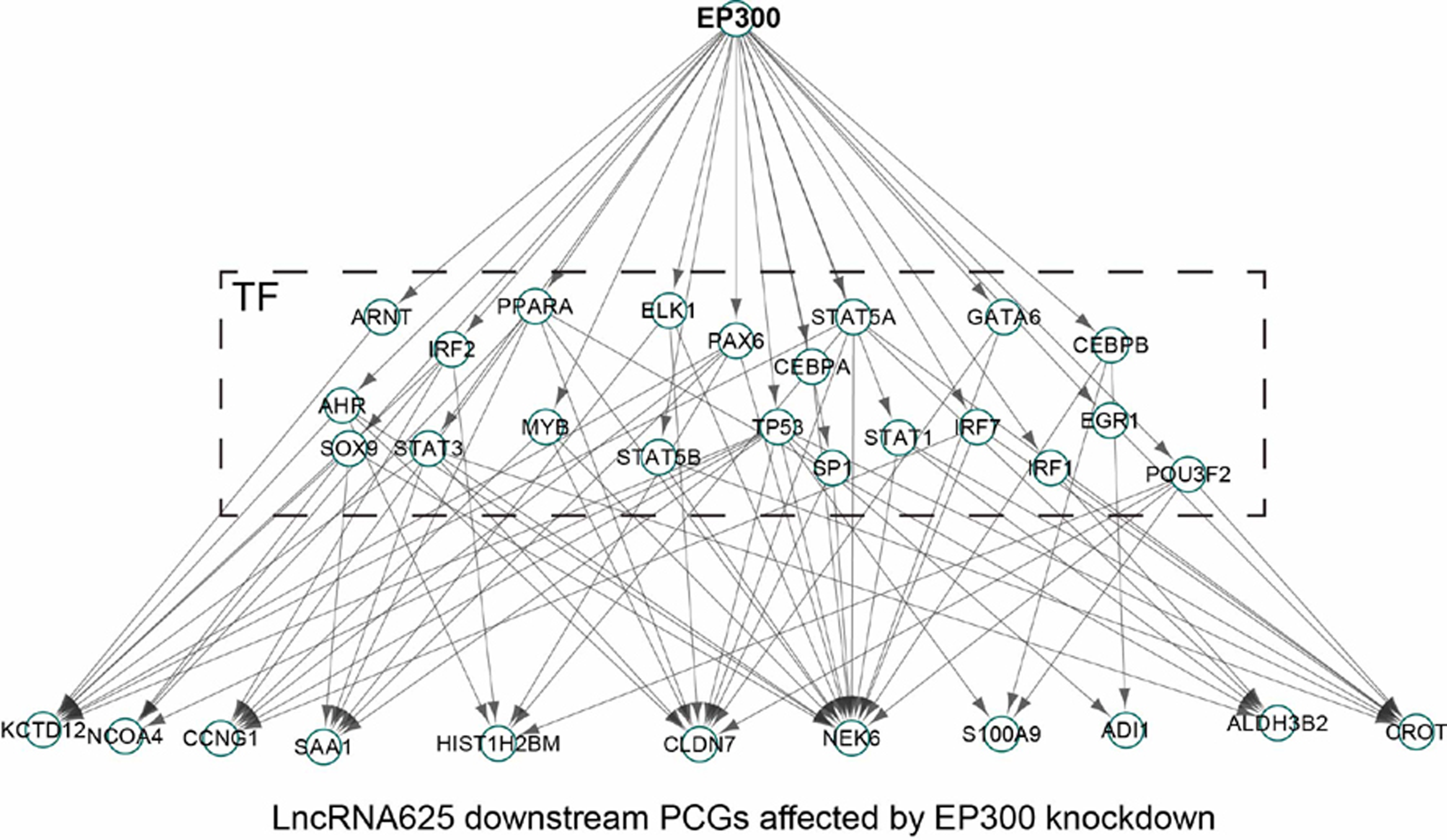

Supplement: Supplementary Figure 8 [file oncsis20171x9.tif]
