# Supplementary material for: Integrative analyses of transcriptome sequencing identify novel functional lncRNAs in esophageal squamous cell carcinoma
Source: Oncogenesis. 2017 Feb 13;6(2):e297–. doi: 10.1038/oncsis.2017.1 (PMC5337622; doi:10.1038/oncsis.2017.1)
Supplement: Supplementary Table 1 [file oncsis20171x10.doc]

**Supplementary Table 1. Clinical characteristics of patients with ESCC for RNA-Seq analysis.**

| **Data set No** | **Data set ID** | **Sex** | **Age** | **pTNM stage** |
| --- | --- | --- | --- | --- |
| 1 | 786N/T | male | 76 | ⅠB |
| 2 | 200N/T | female | 54 | ⅠB |
| 3 | 783N/T | female | 55 | ⅡA |
| 4 | 788N/T | male | 55 | ⅡA |
| 5 | 797N/T | male | 61 | ⅡB |
| 6 | 798N/T | male | 49 | ⅡB |
| 7 | 799N/T | female | 73 | ⅡB |
| 8 | 801N/T | female | 56 | ⅡB |
| 9 | 199N/T | male | 64 | ⅡB |
| 10 | 785N/T | male | 49 | ⅢA |
| 11 | 804N/T | male | 55 | ⅢA |
| 12 | 791N/T | male | 45 | ⅢA |
| 13 | 794N/T | male | 49 | ⅢA |
| 14 | 782N/T | male | 51 | ⅢB |
| 15 | 800N/T | male | 56 | ⅢB |

T: present tumor; N: present adjacent non-tumor tissue
