# Supplementary material for: Integrative analyses of transcriptome sequencing identify novel functional lncRNAs in esophageal squamous cell carcinoma
Source: Oncogenesis. 2017 Feb 13;6(2):e297–. doi: 10.1038/oncsis.2017.1 (PMC5337622; doi:10.1038/oncsis.2017.1)
Supplement: Supplementary Table 2 [file oncsis20171x11.doc]

**Table S2. RNA sequencing information of 15 paired ESCC and non-tumor tissues**

a:there are two read fastq files for paired-end RNA-seq data for each sample.

| **Supplementary Table 2. Summary of RNA-seq in 30 esophageal samples.** | | | |
| --- | --- | --- | --- |
| **Sample No.** | **Size of Fastq file 1 (kb)^a^** | **Size of Fastq file 2 (kb)^a^** | **Mapped read number^b^** |
| B799T | 5327114 | 5327114 | 48709263 |
| B786N | 4977788 | 4977788 | 46632908 |
| B786T | 5206514 | 5206514 | 49494504 |
| B788T | 4927479 | 4927479 | 48775449 |
| C200T | 4993606 | 4993606 | 50748681 |
| B791T | 5029657 | 5029657 | 47296559 |
| B797T | 5119423 | 5119423 | 47689704 |
| B782T | 5146246 | 5146246 | 48794226 |
| C200N | 5479234 | 5479234 | 53725830 |
| B794N | 5550207 | 5550207 | 54131355 |
| B783T | 5615038 | 5615038 | 51993543 |
| B788N | 5648280 | 5648280 | 55663521 |
| B783N | 5805026 | 5805026 | 56331701 |
| B801N | 5811819 | 5811819 | 59084391 |
| B785N | 6087885 | 6087885 | 61159267 |
| B800T | 6165448 | 6165448 | 57516176 |
| B797N | 6218617 | 6218617 | 60473947 |
| B804T | 6369044 | 6369044 | 61009343 |
| B799N | 6422506 | 6422506 | 67008508 |
| B791N | 6497211 | 6497211 | 64658410 |
| B800N | 6711548 | 6711548 | 66182717 |
| B801T | 6869029 | 6869029 | 68270422 |
| B804N | 6913678 | 6913678 | 73827584 |
| B785T | 7022047 | 7022047 | 69984057 |
| B794T | 7043444 | 7043444 | 67377173 |
| C199N | 7590629 | 7590629 | 69289494 |
| B798N | 8196458 | 8196458 | 80657184 |
| B798T | 8276697 | 8276697 | 79038486 |
| C199T | 9088269 | 9088269 | 78104548 |
| B782N | 13231971 | 13231971 | 125607644 |
| T: tumor tissues; N: non-tumor tissues | | | |

b: The number of reads mapped to known genes for each sample after performing easyRNAseq.
