# Supplementary material for: Integrative analyses of transcriptome sequencing identify novel functional lncRNAs in esophageal squamous cell carcinoma
Source: Oncogenesis. 2017 Feb 13;6(2):e297–. doi: 10.1038/oncsis.2017.1 (PMC5337622; doi:10.1038/oncsis.2017.1)
Supplement: Supplementary Table 3 [file oncsis20171x12.doc]

**Supplementary Table 3. The statistically significant functional lncRNAs identified by URW-LPE**

| **LncRNA ID** | **LncRNA names** | **URWScore** | ***P*-value** | **FDR** | **Rank**  **(URWScore)** | **Rank**  **(FC)** |
| --- | --- | --- | --- | --- | --- | --- |
| **ENSG00000258294** | **RP11-314D7.1.1** | **0.615425989** | **1.13821E-05** | **0.007** | **1** | **1** |
| **ENSG00000184324** | **ENSG00000184324** | **0.606154633** | **2.60163E-05** | **0.0077** | **2** | **88** |
| **ENSG00000259756** | **RP11-625H11.2.1** | **0.597015104** | **4.71545E-05** | **0.0077** | **3** | **1** |
| **ENSG00000261319** | **RP11-279O17.1.1** | **0.593324313** | **5.36585E-05** | **0.0077** | **4** | **1** |
| **ENSG00000229876** | **RP11-199O14.1.1** | **0.590463778** | **6.26016E-05** | **0.0077** | **5** | **1** |
| **ENSG00000250735** | **RP11-567N4.3.1** | **0.577663659** | **0.000113008** | **0.011583333** | **6** | **1** |
| **ENSG00000250252** | **RP11-342A1.1.1** | **0.567272038** | **0.000181301** | **0.01325** | **7** | **1** |
| **ENSG00000250708** | **RP11-161D15.1.1** | **0.564603831** | **0.000200813** | **0.01325** | **8** | **1** |
| **ENSG00000248268** | **CTC-499J9.1.1** | **0.564269955** | **0.000203252** | **0.01325** | **9** | **1** |
| **ENSG00000248370** | **RP11-366H4.1.1** | **0.562804035** | **0.000215447** | **0.01325** | **10** | **1** |
| **ENSG00000223812** | **RP11-197K6.1.1** | **0.556142667** | **0.000279675** | **0.014576923** | **11** | **1** |
| **ENSG00000250920** | **RP11-297P16.4.1** | **0.553914404** | **0.00030813** | **0.014576923** | **12** | **1** |
| **ENSG00000251577** | **RP11-297P16.3.1** | **0.553914404** | **0.00030813** | **0.014576923** | **12** | **1** |
| **ENSG00000257114** | **RP11-25I15.3.1** | **0.54561862** | **0.000420325** | **0.017055556** | **14** | **1** |
| **ENSG00000236289** | **AC130710.1.1** | **0.542755874** | **0.000477236** | **0.017055556** | **15** | **78** |
| **ENSG00000254519** | **CTD-2210P24.1.1** | **0.542734828** | **0.000477236** | **0.017055556** | **16** | **1** |
| **ENSG00000197463** | **ENSG00000197463** | **0.542361568** | **0.000482114** | **0.017055556** | **17** | **89** |
| **ENSG00000251185** | **RP11-542G1.1.1** | **0.541249601** | **0.000499187** | **0.017055556** | **18** | **1** |
| **ENSG00000255426** | **CTD-2210P24.2.1** | **0.535984954** | **0.000630894** | **0.020068182** | **19** | **1** |
| **ENSG00000235523** | **RP11-63P12.7.1** | **0.534726024** | **0.000664228** | **0.020068182** | **20** | **1** |
| **ENSG00000230316** | **RP11-560I19.4.1** | **0.533408636** | **0.000704065** | **0.020068182** | **21** | **127** |
| **ENSG00000233085** | **XXyac-YX65C7_A.3.1** | **0.532810445** | **0.000717886** | **0.020068182** | **22** | **153** |
| **ENSG00000253659** | **RP11-1114I9.1.1** | **0.531564633** | **0.000762602** | **0.020391304** | **23** | **1** |
| **ENSG00000228952** | **RP11-567G11.1.1** | **0.528247287** | **0.000869106** | **0.021314815** | **24** | **168** |
| **ENSG00000259631** | **RP11-557C18.4.1** | **0.52726803** | **0.000904878** | **0.021314815** | **25** | **1** |
| **ENSG00000228295** | **LINC00392** | **0.526646553** | **0.000928455** | **0.021314815** | **26** | **1** |
| **ENSG00000215115** | **ENSG00000215115** | **0.526407413** | **0.000935772** | **0.021314815** | **27** | **85** |
| **ENSG00000183242** | **WT1-AS** | **0.523871125** | **0.001035772** | **0.022224138** | **28** | **1** |
| **ENSG00000246350** | **RP5-1186N24.3.1** | **0.523607782** | **0.001047967** | **0.022224138** | **29** | **1** |
| **ENSG00000261807** | **RP11-430C1.1.1** | **0.521221509** | **0.001147154** | **0.023016129** | **30** | **1** |
| **ENSG00000231764** | **DLX6-AS1** | **0.52092643** | **0.001160163** | **0.023016129** | **31** | **199** |
| **ENSG00000243321** | **RP11-167H9.3.1** | **0.516920516** | **0.001365041** | **0.02555** | **32** | **1** |
| **ENSG00000263677** | **ENSG00000263677** | **0.515686182** | **0.001422764** | **0.02555** | **33** | **78** |
| **ENSG00000250564** | **RP11-215P8.4.1** | **0.514711239** | **0.00149187** | **0.02555** | **34** | **1** |
| **ENSG00000228630** | **HOTAIR** | **0.514190412** | **0.001519512** | **0.02555** | **35** | **93** |
| **ENSG00000215113** | **ENSG00000215113** | **0.514149436** | **0.001521951** | **0.02555** | **36** | **84** |
| **ENSG00000235086** | **FNDC1-IT1** | **0.51222982** | **0.001635772** | **0.02555** | **37** | **1** |
| **ENSG00000225107** | **AC092484.1.1** | **0.51210113** | **0.001643902** | **0.02555** | **38** | **1** |
| **ENSG00000233338** | **TLR8-AS1** | **0.51199399** | **0.001647967** | **0.02555** | **39** | **1** |
| **ENSG00000259811** | **RP11-863P13.3.1** | **0.511793592** | **0.001661789** | **0.02555** | **40** | **239** |
| **ENSG00000233850** | **AC103563.8.1** | **0.509437508** | **0.001814634** | **0.026845238** | **41** | **143** |
| **ENSG00000256513** | **RP11-977P2.1.1** | **0.509185802** | **0.001833333** | **0.026845238** | **42** | **78** |
| **ENSG00000233532** | **LINC00460** | **0.503077484** | **0.002284553** | **0.032674419** | **43** | **130** |
| **ENSG00000233358** | **RP1-209A6.1.1** | **0.498816389** | **0.002673171** | **0.037363636** | **44** | **1** |
| **ENSG00000225226** | **AC007250.4.1** | **0.496587697** | **0.002895935** | **0.039577778** | **45** | **1** |
| **ENSG00000259410** | **RP11-34F13.3.1** | **0.495422899** | **0.003025203** | **0.039914894** | **46** | **1** |
| **ENSG00000204460** | **AC079586.1.1** | **0.495189982** | **0.003050407** | **0.039914894** | **47** | **121** |
| **ENSG00000263655** | **ENSG00000263655** | **0.494120329** | **0.003189431** | **0.040460784** | **48** | **1** |
| **ENSG00000235578** | **XXbac-B33L19.4.1** | **0.493556849** | **0.003244715** | **0.040460784** | **49** | **1** |
| **ENSG00000205634** | **RP11-191L9.6.1** | **0.492502966** | **0.003355285** | **0.040460784** | **50** | **98** |
| **ENSG00000258763** | **RP11-110A12.2.1** | **0.492501252** | **0.003355285** | **0.040460784** | **51** | **1** |
| **ENSG00000266869** | **ENSG00000266869** | **0.490917075** | **0.003555285** | **0.041425926** | **52** | **1** |
| **ENSG00000229618** | **AC011288.2.1** | **0.490681911** | **0.003586179** | **0.041425926** | **53** | **119** |
| **ENSG00000244791** | **RP11-65D17.1.1** | **0.490310607** | **0.003637398** | **0.041425926** | **54** | **1** |
| **ENSG00000253661** | **RP11-65D13.1.1** | **0.488028338** | **0.003926829** | **0.043719298** | **55** | **112** |
| **ENSG00000249413** | **RP11-25H12.1.1** | **0.487414096** | **0.004001626** | **0.043719298** | **56** | **1** |
| **ENSG00000260115** | **CTC-420A11.2.1** | **0.487063098** | **0.004052033** | **0.043719298** | **57** | **1** |
| **ENSG00000258657** | **RP11-104E19.1.1** | **0.486178135** | **0.004174797** | **0.044267241** | **58** | **129** |
| **ENSG00000230838** | **AC093850.2.1** | **0.485158292** | **0.004334959** | **0.0445** | **59** | **103** |
| **ENSG00000243810** | **RP5-1096J16.1.1** | **0.485129068** | **0.004341463** | **0.0445** | **60** | **1** |
| **ENSG00000265579** | **ENSG00000265579** | **0.483202017** | **0.004645528** | **0.046836066** | **61** | **287** |
| **ENSG00000250590** | **RP11-565A3.2.1** | **0.482796724** | **0.004723577** | **0.046854839** | **62** | **1** |
| **ENSG00000254417** | **ANO1-AS2** | **0.480855496** | **0.005035772** | **0.04915873** | **63** | **150** |
| ENSG00000229732 | AC019349.5.1 | 0.478852451 | 0.005362602 | 0.051453846 | 64 | 174 |
| ENSG00000236212 | AC009262.2.1 | 0.478538298 | 0.005438211 | 0.051453846 | 65 | 1 |
| ENSG00000258188 | RP11-146E13.4.1 | 0.477242206 | 0.00565935 | 0.052734848 | 66 | 1 |
| ENSG00000251151 | AC012531.14.1 | 0.476480425 | 0.00581626 | 0.052956522 | 67 | 94 |
| ENSG00000244383 | RP11-475O23.3.1 | 0.476087598 | 0.005886992 | 0.052956522 | 68 | 227 |
| ENSG00000240922 | LSAMP-AS1 | 0.475778012 | 0.005941463 | 0.052956522 | 69 | 120 |
| ENSG00000226053 | RP5-1070A16.1.1 | 0.474797377 | 0.006130894 | 0.053864286 | 70 | 1 |
| ENSG00000226725 | RP11-493K23.1.1 | 0.473561583 | 0.006378862 | 0.055150685 | 71 | 111 |
| ENSG00000233589 | RP4-694A7.2.1 | 0.473130573 | 0.006472358 | 0.055150685 | 72 | 376 |
| ENSG00000259974 | LINC00261 | 0.472759214 | 0.006546341 | 0.055150685 | 73 | 107 |
| ENSG00000254282 | RP11-373N22.4.1 | 0.472085166 | 0.006680488 | 0.055173333 | 74 | 100 |
| ENSG00000232325 | AC093627.7.1 | 0.471866743 | 0.006728455 | 0.055173333 | 75 | 1 |
| ENSG00000231963 | RP11-493K23.4.1 | 0.470850782 | 0.006961789 | 0.055467949 | 76 | 101 |
| ENSG00000266560 | ENSG00000266560 | 0.470550642 | 0.007030081 | 0.055467949 | 77 | 116 |
| ENSG00000226070 | RP11-552E20.4.1 | 0.470533094 | 0.007034959 | 0.055467949 | 78 | 78 |
| ENSG00000226363 | AC009336.24.1 | 0.469447622 | 0.007274797 | 0.056632911 | 79 | 180 |
| ENSG00000253394 | LINC00534 | 0.468309253 | 0.007587805 | 0.058 | 80 | 1 |
| ENSG00000236651 | AC104801.1.1 | 0.467858481 | 0.007703252 | 0.058 | 81 | 1 |
| ENSG00000258240 | RP1-46F2.3.1 | 0.467733682 | 0.007733333 | 0.058 | 82 | 1 |
| ENSG00000253892 | RP11-770E5.2.1 | 0.466509825 | 0.008017886 | 0.059409639 | 83 | 1 |
| ENSG00000258331 | RP11-118A3.1.1 | 0.466094314 | 0.008134146 | 0.059553571 | 84 | 146 |
| ENSG00000110347 | ENSG00000110347 | 0.464254589 | 0.008635772 | 0.060858696 | 85 | 126 |
| ENSG00000250777 | RP13-884E18.4.1 | 0.464074709 | 0.008686992 | 0.060858696 | 86 | 1 |
| ENSG00000253508 | RP1-170O19.14.1 | 0.464029447 | 0.008698374 | 0.060858696 | 87 | 1 |
| ENSG00000251361 | CTD-2091N23.1.1 | 0.463625305 | 0.008804065 | 0.060858696 | 88 | 1 |
| ENSG00000231689 | AC068718.1.1 | 0.463455705 | 0.008846341 | 0.060858696 | 89 | 248 |
| ENSG00000248112 | RP11-78C3.1.1 | 0.463048781 | 0.008976423 | 0.060858696 | 90 | 1 |
| ENSG00000227400 | AC012501.2.1 | 0.462892509 | 0.009020325 | 0.060858696 | 91 | 1 |
| ENSG00000233515 | AL022344.4.1 | 0.462609164 | 0.009104065 | 0.060858696 | 92 | 1 |
| ENSG00000233078 | RP11-5P18.5.1 | 0.461648136 | 0.009378862 | 0.061947917 | 93 | 117 |
| ENSG00000265069 | ENSG00000265069 | 0.461327972 | 0.009484553 | 0.061947917 | 94 | 181 |
| ENSG00000265702 | ENSG00000265702 | 0.460727576 | 0.009666667 | 0.061947917 | 95 | 305 |
| ENSG00000253554 | RP11-32K4.1.1 | 0.460714539 | 0.009669919 | 0.061947917 | 96 | 1 |
| ENSG00000255921 | RP11-662I13.2.1 | 0.456628502 | 0.010926016 | 0.069273196 | 97 | 1 |
| ENSG00000258927 | RP11-1070N10.5.1 | 0.456167852 | 0.011095122 | 0.069627551 | 98 | 1 |
| ENSG00000230410 | XXbac-B33L19.6.1 | 0.45563664 | 0.011270732 | 0.06983 | 99 | 1 |
| ENSG00000256321 | RP11-153K16.1.1 | 0.455386055 | 0.011354472 | 0.06983 | 100 | 1 |
| ENSG00000242147 | RP13-463N16.6.1 | 0.45416428 | 0.011773171 | 0.071117647 | 101 | 118 |
| ENSG00000249001 | RP11-742B18.1.1 | 0.454114991 | 0.011795122 | 0.071117647 | 102 | 1 |
| ENSG00000259107 | CTD-2128A3.1.1 | 0.453371803 | 0.012091057 | 0.071316038 | 103 | 185 |
| ENSG00000264660 | ENSG00000264660 | 0.453131198 | 0.012193496 | 0.071316038 | 104 | 140 |
| ENSG00000236908 | RP5-1063M23.2.1 | 0.452899505 | 0.01227561 | 0.071316038 | 105 | 321 |
| ENSG00000225062 | AC021016.6.1 | 0.452866067 | 0.01229187 | 0.071316038 | 106 | 434 |
| ENSG00000254605 | RP11-626H12.2.1 | 0.452365404 | 0.012463415 | 0.071635514 | 107 | 176 |
| ENSG00000260922 | RP11-538I12.3.1 | 0.451672332 | 0.01274065 | 0.072077982 | 108 | 242 |
| ENSG00000228189 | RP13-60M5.2.1 | 0.451548741 | 0.012774797 | 0.072077982 | 109 | 1 |
| ENSG00000227964 | RP5-1112F19.2.1 | 0.450955332 | 0.012979675 | 0.072568182 | 110 | 1 |
| ENSG00000234919 | AC064834.3.1 | 0.45028167 | 0.013249593 | 0.07340991 | 111 | 1 |
| ENSG00000250697 | CTD-2066L21.3.1 | 0.449880553 | 0.013397561 | 0.073565789 | 112 | 141 |
| ENSG00000237361 | RP11-269C23.3.1 | 0.449531299 | 0.013549593 | 0.073565789 | 113 | 220 |
| ENSG00000229243 | AC098973.1.1 | 0.449320778 | 0.013636585 | 0.073565789 | 114 | 184 |
| ENSG00000244040 | CTD-2049J23.2.1 | 0.448533217 | 0.013965854 | 0.074439655 | 115 | 147 |
| ENSG00000259672 | RP11-69G7.1.1 | 0.448353087 | 0.01404065 | 0.074439655 | 116 | 83 |
| ENSG00000224750 | RP11-94M14.2.1 | 0.447889356 | 0.014230081 | 0.074799145 | 117 | 1 |
| ENSG00000237265 | RP11-402P6.9.1 | 0.447365989 | 0.014470732 | 0.075419492 | 118 | 115 |
| ENSG00000236117 | RP4-754E20__A.5.1 | 0.446943668 | 0.014665041 | 0.075789916 | 119 | 1 |
| ENSG00000260372 | CHST9-AS1 | 0.446568298 | 0.014829268 | 0.076 | 120 | 292 |
| ENSG00000225548 | AC098973.2.1 | 0.44549127 | 0.01526748 | 0.077599174 | 121 | 1 |
| ENSG00000249641 | AC012531.10.1 | 0.444990825 | 0.015495935 | 0.078114754 | 122 | 108 |
| ENSG00000225087 | RP4-660H19.1.1 | 0.44422229 | 0.015835772 | 0.078736 | 123 | 131 |
| ENSG00000230725 | RP4-738P15.1.1 | 0.444008246 | 0.015936585 | 0.078736 | 124 | 1 |
| ENSG00000230061 | AP001065.2.1 | 0.443850982 | 0.016003252 | 0.078736 | 125 | 250 |
| ENSG00000225146 | AC073957.15.1 | 0.442965896 | 0.016425203 | 0.080170635 | 126 | 109 |
| ENSG00000242781 | RP11-47P18.2.1 | 0.442287745 | 0.016774797 | 0.081232283 | 127 | 1 |
| ENSG00000257002 | AP000438.2.1 | 0.441996364 | 0.016908943 | 0.081242188 | 128 | 217 |
| ENSG00000234921 | RP11-85O21.5.1 | 0.441575498 | 0.017121951 | 0.081627907 | 129 | 139 |
| ENSG00000228437 | RP11-400N13.2.1 | 0.441024273 | 0.017360163 | 0.082126923 | 130 | 82 |
| ENSG00000235385 | GS1-600G8.5.1 | 0.440365822 | 0.017719512 | 0.083187023 | 131 | 1 |
| ENSG00000234948 | RP4-723E3.1.1 | 0.439139263 | 0.018358537 | 0.085296992 | 132 | 1 |
| ENSG00000204044 | RP11-465L10.10.1 | 0.438922242 | 0.018446341 | 0.085296992 | 133 | 125 |
| ENSG00000258670 | RP11-1042B17.3.1 | 0.437856387 | 0.019026016 | 0.087320896 | 134 | 122 |
| ENSG00000265380 | ENSG00000265380 | 0.437579296 | 0.019178049 | 0.087366667 | 135 | 1 |
| ENSG00000240661 | RP11-174O3.3.1 | 0.436046925 | 0.020056098 | 0.090083942 | 136 | 1 |
| ENSG00000253675 | CTD-3118D11.2.1 | 0.43602958 | 0.02006748 | 0.090083942 | 137 | 110 |
| ENSG00000231453 | AC018470.4.1 | 0.435418892 | 0.020426016 | 0.090661871 | 138 | 315 |
| ENSG00000253477 | RP11-1C8.4.1 | 0.435297979 | 0.020491057 | 0.090661871 | 139 | 162 |
| ENSG00000251281 | CTD-2066L21.2.1 | 0.434778582 | 0.020792683 | 0.091339286 | 140 | 136 |
| ENSG00000232900 | RP4-697P8.3.1 | 0.434127074 | 0.021162602 | 0.092304965 | 141 | 235 |
| ENSG00000233052 | RP11-398B16.2.1 | 0.432771574 | 0.02200813 | 0.095316901 | 142 | 270 |
| ENSG00000233485 | RP3-467K16.2.1 | 0.431146474 | 0.023055285 | 0.098144828 | 143 | 341 |
| ENSG00000248554 | RP11-159F24.6.1 | 0.431029542 | 0.023136585 | 0.098144828 | 144 | 284 |
| ENSG00000265369 | ENSG00000265369 | 0.431024286 | 0.023139837 | 0.098144828 | 145 | 152 |
| ENSG00000224049 | RP11-30O15.1.1 | 0.430309516 | 0.023646341 | 0.099367347 | 146 | 1 |
| ENSG00000232243 | LINC00414 | 0.430128152 | 0.02375122 | 0.099367347 | 147 | 1 |
