# Supplementary material for: Integrative analyses of transcriptome sequencing identify novel functional lncRNAs in esophageal squamous cell carcinoma
Source: Oncogenesis. 2017 Feb 13;6(2):e297–. doi: 10.1038/oncsis.2017.1 (PMC5337622; doi:10.1038/oncsis.2017.1)
Supplement: Supplementary Table 4 [file oncsis20171x13.doc]

**Supplementary Table 4. Clinicopathological characteristics of patients with ESCC for qRT-PCR.**

| **Clinical and pathological indexes** | **Case No.** | **5-year OS (%)** | **P*** | **5-year DFS(%)** | **P*** |
| --- | --- | --- | --- | --- | --- |
| Specimens | 120 |  |  |  |  |
| Mean age | 58.21 |  |  |  |  |
| Age (years) |  |  |  |  |  |
| <58 | 61 | 62.6 | 0.015 | 61.0 | 0.040 |
| ≥58 | 59 | 43.2 |  | 40.7 |  |
| Gender |  |  |  |  |  |
| Female | 30 | 56.7 | 0.917 | 46.7 | 0.625 |
| Male | 90 | 51.6 |  | 51.2 |  |
| Tumor size |  |  |  |  |  |
| ≤3cm | 34 | 56.9 | 0.571 | 56.6 | 0.446 |
| 3-5cm | 60 | 53.7 |  | 51.6 |  |
| 5cm | 26 | 44.2 |  | 39.7 |  |
| Tumor location |  |  |  |  |  |
| upper | 11 | 44.5 | 0.559 | 43.6 | 0.453 |
| middle | 40 | 49.5 |  | 46.1 |  |
| lower | 69 | 56.1 |  | 53.0 |  |
| Histologic grade |  |  |  |  |  |
| G1 | 20 | 62.9 | 0.044 | 62.6 | 0.056 |
| G2 | 90 | 53.3 |  | 49.2 |  |
| G3 | 10 | 20.0 |  | 20.0 |  |
| Invasive depth |  |  |  |  |  |
| T1 | 7 | 100.0 | 0.026 | 57.1 | 0.202 |
| T2 | 15 | 33.3 |  | 33.3 |  |
| T3 | 97 | 53.0 |  | 52.9 |  |
| T4 | 1 | 0.0 |  | 0.0 |  |
| Lymph node metastasis |  |  |  |  |  |
| N0 | 67 | 65.6 | 0.000 | 64.1 | 0.001 |
| N1 | 37 | 47.5 |  | 38.6 |  |
| N2 | 10 | 10.0 |  | 11.7 |  |
| N3 | 6 | 0.0 |  | 0.0 |  |
| pTNM-stage |  |  |  |  |  |
| I | 14 | 77.9 | 0.001 | 71.4 | 0.037 |
| II | 59 | 61.0 |  | 57.5 |  |
| III | 47 | 33.4 |  | 33.2 |  |

* Kaplan-Meier log-rank test; P <0.05 was considered significant. All patients underwent surgical treatment. OS: overall survival; DFS: disease-free survival
