# Supplementary material for: Integrative analyses of transcriptome sequencing identify novel functional lncRNAs in esophageal squamous cell carcinoma
Source: Oncogenesis. 2017 Feb 13;6(2):e297–. doi: 10.1038/oncsis.2017.1 (PMC5337622; doi:10.1038/oncsis.2017.1)
Supplement: Supplementary Table 5 [file oncsis20171x14.docx]

| Gene | Forward (5’ to 3’) | Reverse (5’ to 3’) |
| --- | --- | --- |
| lncRNA625 | AGAGACCACCATCAAGGGATAAAAT | GGCTAATAAACAGGGTCTTCAGGT |
| LINC00460 | ATTGTGTGGGAGGCGTCTGTGTAGC | TTTGGGTGGGGGACATAAACATTCG |
| AC093850.2 | AGAGAGGATTCCACTGCGTGTATGT | CTGGGAGGAAGGAACATTGAAGTCT |
| RP1-151B14.6 | CAGACCAAGGAAAGGCAGCCGAGAC | GTGGCATCTACTTCTTCAGCCTCAA |
| RP11-107M16.2 | GTTCCTGATACTGGTTTTTCTACAT | TTTTATTCTCCAAGGTTGTGCTATG |
| LINC01269 | CTCAAACTCCTGGGCTCAAGAAATC | CCATCCCTAAGAATACCCCCACAGT |
| RP11-417E7.1 | GAAAAACAGTGAAGTTGAGGTGAGC | GGGGATTAGGGCTTCAACTTAGGGC |
| RP11-435D7.3 | CTTCCCACATCACTTCCTGACACCT | GAATGAGGTATTTGGGCAGATGTTT |
| TNC | TGACAGAAGTGACGGAAGAGACGGT | CAGGATGGCAAATACACGGATAAAG |
| NEK6 | GCCTCTTGAAGCAACTGAACCACCC | ATCATCTGCGAGAGGTCCCCTGCGT |
| S100A9 | TCAACACCTTCCACCAATACTCTG | GACCTTTTCATTCTTATTCTCCTTC |
| CLDN7 | GAGCCCTAATGGTGGTCTCCCTGGT | TTCTTCACTTTGTCGTCTCCCCCAC |
| SERINB1 | GAAGTGAATGAAGAGGGAACAGAGG | CCGAATAAAGAAAAGGAATGGATGG |
| GLT25D1 | GAAACACTGGTCTGACTCACGCTAC | CTTGTAGTAGCCCTGGGAAGTCATT |
| HIST1H2BM | CAGTCAAATCTGCTCCAGTCCCTAA | CGCTTCTTTCCATCCTTCTTCTGAG |
| CROT | AGTGGTGGCTGAATGTTGCCTATCT | GCTGCCAGTAGTTCAAGTTATGCCA |
| ALDH3B2 | CAGAGAAGGTCCTGGCTGAGGTGCT | GCTCCCTGTGAAGAAGATGTAGTCC |
| ADI1 | GATGTGAGGGACAAGGAGGACCAGT | GCCTTCGTGTAGTTCTTCTCGTCCA |
| HMGCS1 | GGATGGACGGTATGCCCTGGTAGTT | GCACTGAGGTAGCACTGTATGGAGA |
| KCTD12 | TGGGGACACCCTGAACGAAAGCC | ATCTTGTCCTCGCTCTGGTCGGTGC |
| NCOA4 | AAACCAGCCCAAAGGTGTGGAGATT | CTGGACAAGCCAATCCTCTGTAACC |
| TMPRSS4 | TTGGCAACTTCACTTGTAGGGCTGT | GCTGCTGCCTCTCTCTGTGTATTCC |
| SWAP70 | GGAGAAGCAGGAGTTGGAGAATCAG | CTTGGTCTTATTAGTTGCCATCTCC |
| SAA1 | GTTTTCTGCTCCTTGGTCCTGGGTG | TGGAAGTATTTGTCTGAGCCGATGT |
| TLN2 | CAGCCGAACATTCAGAGAACAAGGA | TCTGGGCTTGAAATCCACCAAACTC |
| U6 | CTCGCTTCGGCAGCACA | AACGCTTCACGAATTTGCGT |
| GAPDH | AGAACATCATCCCTGCCTCTACTGG | CCTGCTTCACCACCTTCTTGATGTC |
| β-actin | AGCGAGCATCCCCCAAAGTT | GGGCACGAAGGCTCATCATT |
| lncRNA625 (full length) | CCCAAGCTTACATCTAGGAAGTGAG-AAGCGTCTC | AAGGAAAAAAGCGGCCGCAAAAACAC-CAGAGAGGGCATTCGGA |

**Supplementary Table 5. Primer sequences for PCR and real-time RT-PCR.**
