# Supplementary material for: Integrative analyses of transcriptome sequencing identify novel functional lncRNAs in esophageal squamous cell carcinoma
Source: Oncogenesis. 2017 Feb 13;6(2):e297–. doi: 10.1038/oncsis.2017.1 (PMC5337622; doi:10.1038/oncsis.2017.1)
Supplement: Supplementary Table 6 [file oncsis20171x15.doc]

**Supplementary Table 6. EP300 Chip-seq data information of cell lines obtained from UCSC databases.**

| **Short Label** | **Description** | [**Antibody or target protein**](http://genome.ucsc.edu/cgi-bin/hgEncodeVocab?type=) | **Long Label** | [**UCSC Accession**](http://genome.ucsc.edu/cgi-bin/hgEncodeVocab?type=) | **Table name** |
| --- | --- | --- | --- | --- | --- |
| [H1-hESC EP300](http://genome.ucsc.edu/cgi-bin/hgTrackUi?hgsid=442560251_ZFEN6lstdpmJS1spPTO9i7PB8Q5G&g=wgEncodeAwgTfbsHaibH1hescP300V0416102UniPk) | Embryonic stem cells | [P300/EP300 (sc-585)](http://genome.ucsc.edu/cgi-bin/hgEncodeVocab?term=) | H1-hESC TFBS Uniform Peaks of p300 from ENCODE/HudsonAlpha/Analysis | wgEncodeEH001574 | wgEncodeAwgTfbsHaibH1hescP300V0416102UniPk |
| [HeLa-S3 EP300](http://genome.ucsc.edu/cgi-bin/hgTrackUi?hgsid=442561041_fRk58WcAPm1lMWV75Q48sTl2BgOf&g=wgEncodeAwgTfbsSydhHelas3P300sc584sc584IggrabUniPk) | Cervical carcinoma | [P300/EP300 (sc-584)](http://genome.ucsc.edu/cgi-bin/hgEncodeVocab?term=) | HeLa-S3 TFBS Uniform Peaks of p300_(SC-584) from ENCODE/Stanford/Analysi | wgEncodeEH001820 | wgEncodeAwgTfbsSydhHelas3P300sc584sc584IggrabUniPk |
| [HepG2 EP300 h](http://genome.ucsc.edu/cgi-bin/hgTrackUi?hgsid=442560513_7H1FA0MWYRtR67abqot3i3REZjEZ&g=wgEncodeAwgTfbsHaibHepg2P300V0416101UniPk) | Hepatocellular carcinoma | [P300/EP300 (sc-585)](http://genome.ucsc.edu/cgi-bin/hgEncodeVocab?term=) | HepG2 TFBS Uniform Peaks of p300 from ENCODE/HudsonAlpha/Analysis | wgEncodeEH001612 | wgEncodeAwgTfbsHaibHepg2P300V0416101UniPk |
| [HepG2 EP300 s](http://genome.ucsc.edu/cgi-bin/hgTrackUi?hgsid=442560513_7H1FA0MWYRtR67abqot3i3REZjEZ&g=wgEncodeAwgTfbsSydhHepg2P300sc582IggrabUniPk) | Hepatocellular carcinoma | [P300/EP300 (sc-584)](http://genome.ucsc.edu/cgi-bin/hgEncodeVocab?term=) | HepG2 TFBS Uniform Peaks of p300_(SC-584) from ENCODE/Stanford/Analysis | wgEncodeEH001862 | wgEncodeAwgTfbsSydhHepg2P300sc582IggrabUniPk |
| SK-N-SH_RA EP300 | Neuroblastoma cell line, treatment: differentiated with retinoic acid, (Biedler, et al. Morphology and Growth, Tumorigenicity, and Cytogenetics of Human Neuroblastoma Cells in Continuous Culture. Cancer Research 33, 2643-2652, November 1973.) | [P300/EP300 (sc-585)](http://genome.ucsc.edu/cgi-bin/hgEncodeVocab?term=) | SK-N-SH_RA TFBS Uniform Peaks of p300 from ENCODE/HudsonAlpha/Analysis | wgEncodeEH001616 | wgEncodeAwgTfbsHaibSknshraP300V0416102UniPk |
| T-47D+DMSO EP300 | Epithelial cell line derived from a mammary ductal carcinoma | [P300/EP300 (sc-585)](http://genome.ucsc.edu/cgi-bin/hgEncodeVocab?term=) | T-47D (DMSO) TFBS Uniform Peaks of p300 from ENCODE/HudsonAlpha/Analysis | wgEncodeEH001602 | wgEncodeAwgTfbsHaibT47dP300V0416102Dm002p1hUniPk |
| A549+Et.02 EP300 | Epithelial cell line derived from a lung carcinoma tissue. (PMID: 175022), "This line was initiated in 1972 by D.J. Giard, et al. through explant culture of lung carcinomatous tissue from a 58-year-old caucasian male." - ATCC, newly promoted to tier 2: not in 2011 analysis | [P300/EP300 (sc-585)](http://genome.ucsc.edu/cgi-bin/hgEncodeVocab?term=) | TFBS Uniform Peaks of p300 from ENCODE/HudsonAlpha/Analysis | wgEncodeEH002292 | wgEncodeAwgTfbsHaibA549P300V0422111Etoh02UniPk |
