# Supplementary material for: Integrative analyses of transcriptome sequencing identify novel functional lncRNAs in esophageal squamous cell carcinoma
Source: Oncogenesis. 2017 Feb 13;6(2):e297–. doi: 10.1038/oncsis.2017.1 (PMC5337622; doi:10.1038/oncsis.2017.1)
Supplement: Supplementary Table 7 [file oncsis20171x16.doc]

| **Supplementary Table 7. Univariate and multivariate analysis of factors associated with disease-free survival.** | | | | | | | | | |
| --- | --- | --- | --- | --- | --- | --- | --- | --- | --- |
| **Variables** | **Univariate analyses** | | | **Multivariate analyses** | | | | | |
|  | **Sig.*** | **HR** | **95% CI for HR** | **Sig.*** | | **HR** | | **95% CI for HR** | |
| Age  (≥58 vs. <58) | 0.042 | 1.685 | 1.018 – 2.788 | 0.064 | | 1.614 | | 0.972 – 2.679 | |
| Gender  (Female vs. Male) | 0.839 | 0.945 | 0.547 – 1.632 |  | |  | |  | |
| Tumor size | 0.314 |  |  |  | |  | |  | |
| ≤3cm |  | 1.000 | Reference |  | |  | |  | |
| 3-5cm | 0.794 | 1.082 | 0.599 – 1.956 |  |  | |  | |  |
| >5cm | 0.165 | 1.624 | 0.820 – 3.219 |  | |  | |  | |
| pTNM-stage | 0.035 |  |  | 0.028 | |  | |  | |
| I |  | 1.000 | Reference |  | | 1.000 | | Reference | |
| II | 0.239 | 1.875 | 0.659 – 5.337 | 0.268 | | 1.81 | | 0.633 – 5.175 | |
| III | 0.032 | 3.127 | 1.100 – 8.890 | 0.033 | | 3.144 | | 1.099 – 8.995 | |
| lncRNA625  (High vs. Low) | 0.044 | 1.737 | 1.014 – 2.975 | 0.022 | | 1.887 | | 1.097 – 3.246 | |
| *Multivariate analysis, Cox proportional hazards regression model. Variables were adopted for their prognostic significance by univariate analysis. | | | | | | | | | |
